# Navigating infection by pathogenic spirochetes: The host‐bacteria interface at the atomic level

**DOI:** 10.1002/pro.70185

**Published:** 2025-06-22

**Authors:** Libor Hejduk, Norbert Müller, Adriana Rathner, Ján Štěrba, Shang‐Cheng Hung, Chia‐Lin Chyan, Ryan O. M. Rego, Martin Strnad

**Affiliations:** ^1^ Faculty of Science University of South Bohemia Ceske Budejovice Czech Republic; ^2^ Institute of Biochemistry, Johannes Kepler University Linz Linz Austria; ^3^ Genomics Research Center, Academia Sinica Taipei Taiwan; ^4^ Department of Chemistry National Dong Hwa University Shoufeng Hualien County Taiwan; ^5^ Institute of Parasitology Biology Centre ASCR, v.v.i Ceske Budejovice Czech Republic

**Keywords:** adhesins, atomic structure, *Borrelia*, infection, *Leptospira*, lipoproteins, nuclear magnetic resonance, spirochetes, *Treponema*, x‐ray crystallography

## Abstract

Pathogenic spirochetes bind and interact with various host structures and molecules throughout the course of infection. By utilizing their outer surface molecules, spirochetes can effectively modulate their dissemination, interact with immune system regulators, and select specific destination niches within the host. The three‐dimensional structures of multiple spirochetal surface proteins have been elucidated, providing insight into their *modus operandi*. This review focuses on the structural characteristics of these sticky molecules and their functional implications, highlighting how these features contribute to the pathogenicity of spirochetes and their ability to persist in the host and vector environments. Recognizing the structural motifs and ligands to which these important virulence determinants bind could open new avenues for developing strategies to block colonization by spirochetal pathogens.

## 1 INTRODUCTION

Spirochetes include several human pathogenic species responsible for serious diseases such as Lyme disease (LD), relapsing fever (RF), leptospirosis, periodontitis, and syphilis. LD is primarily caused by members of the *Borrelia burgdorferi* sensu lato (*Bb* s.l.) complex, including *B. burgdorferi* sensu stricto (*Bb* s.s.), *Borrelia afzelii*, *Borrelia garinii*, and *Borrelia bavariensis* (Strnad & Rego, 2020). In contrast, RF is caused by a distinct group of *Borrelia* species, such as *Borrelia hermsii* and *Borrelia miyamotoi* (Fukunaga et al., 1995; Schwan et al., 2009). *Treponema denticola* (*Td*) is associated with severe periodontitis, contributing to tissue destruction in the oral cavity (Foschi et al., 2006). *Treponema pallidum* (*Tp*) causes syphilis, a systemic sexually transmitted infection (Radolf et al., 2016), whereas *Leptospira interrogans* is responsible for leptospirosis, a zoonotic disease that can lead to kidney and liver damage (Samrot et al., 2021).

Spirochetal infection and dissemination rely heavily on endocellular flagella, the ability to adhere to host molecules and surfaces, and the ability to avoid clearance by the immune system (Strnad et al., 2023, 2024). Spirochetes exhibit unique characteristics in their outer surface proteins, particularly in the manner in which their adhesive molecules are anchored to the bacterial cell surface. Compared to other Gram‐negative bacteria, where such proteins are often embedded in the outer membrane via complex *β*‐barrel domains (Fairman et al., 2011; Niemann et al., 2004), spirochetes seem to adopt a structurally simpler mechanism. In spirochetes, most adhesins are lipoproteins tethered to the bacterial surface via their N‐terminal regions (Cullen et al., 2004). The lipid membrane anchor of the lipoproteins in *Bb* s.s. is composed of an S‐glycerylcysteine thioether, which is modified by one ester‐linked fatty acid, one short acetyl group and one N‐terminal amide‐bound fatty acid (Beermann et al., 2000). The lipid membrane anchor of spirochetes predominantly contains saturated fatty acids palmitate (C16:0) and, to a lesser extent, stearate (C18:0) (Belisle et al., 1994). The remaining C‐terminal portion of the protein is attached to the cysteine residue in the usual fashion via a peptide bond (Beermann et al., 2000). Overall, the anchoring mechanisms of spirochetal adhesins appear to be minimalistic, largely based on hydrophobic interactions between the two fatty acid chains covalently attached to the protein and the lipid bilayer of the membrane. This presumably not only simplifies their insertion and localization on the bacterial surface but may also confer flexibility and mobility to the adhesins within the membrane, allowing spirochetes to dynamically adapt to various environments in their hosts (Strnad et al., 2023).

Outer surface molecules are the first to encounter the host environment, facilitate interactions, and shield pathogens from the host immune system. If this first line of defense is compromised or the correct set of proteins is not present on the bacterial surface, the pathogen is eliminated (Bankhead & Chaconas, 2007; Grimm et al., 2004; Kraiczy et al., 2003). Spirochetes express various surface proteins that bind multiple extracellular matrix (ECM) components. *Bb* s.l. produces proteins like DbpA, DbpB, BBK32, RevA, and OspC, which support host interaction (Brissette, Bykowski, et al., 2009; Fischer et al., 2006; Guo et al., 1995; Lin et al., 2020). In *Td*, Msp binds fibronectin, enhancing tissue adherence and virulence (Edwards et al., 2005). *Tp* uses proteins such as Tp0751 for tissue colonization (Cameron et al., 2008). In *Leptospira* spp., LigA and LigB play similar roles in ECM protein binding, contributing to their persistence in host tissues (Choy et al., 2007; Hoke et al., 2008). Spirochetes have also evolved surface proteins that interact with immune regulators such as factor H (FH) to evade complement‐mediated killing. In *Bb* s.l., proteins such as complement regulator‐acquiring surface proteins (CRASPs) and Erp (OspE‐related) bind to FH, enhancing complement inhibition and immune evasion (Brissette, Haupt, et al., 2009; Kraiczy et al., 2003). Similarly, *Td* employs proteins such as FhbB to recruit host complement regulators (McDowell et al., 2007). *Leptospira* expresses LfhA, which binds FH and its related molecules (Verma et al., 2006). The ability to evade the immune system is crucial for the persistence of spirochetes within the host, which can lead to chronic infections in certain cases (Wong et al., 2022).

Although significant progress has been made in understanding the biological roles of spirochete surface proteins, a complete mechanistic picture, including their intricate structural and functional details, remains largely unknown. Resolving the precise three‐dimensional architecture of virulence‐associated proteins down to the atomic level is crucial for elucidating their functional mechanisms. This includes identifying active sites, characterizing conformational changes during molecular interactions, and understanding how these proteins contribute to pathogenicity. Identifying common binding motifs within these proteins allows researchers to uncover conserved sequences or structural elements that are critical for molecular interactions (Arora et al., 2021; Lilic et al., 2006). High‐resolution structures can reveal previously unrecognized binding sites, thereby opening new avenues for drug discovery (Wells & McClendon, 2007). Moreover, the surface‐exposed nature of these molecules makes them highly accessible for therapeutic targeting. Structural information also serves as a valuable resource for developing diagnostic tools capable of detecting specific virulence factors (Kuhnert et al., 2000).

X‐ray crystallography and nuclear magnetic resonance (NMR) spectroscopy are complementary technologies that can provide atomic‐resolution information on molecular entities. Both can be used to determine three‐dimensional structures of rigid molecules. X‐ray diffraction accounts for the majority of protein structures deposited in the Protein Data Bank (PDB) (Otun & Achilonu, 2023). For the application of x‐ray crystallography, the availability of single crystals is a prerequisite. Diffraction maps of x‐rays yield electron‐density maps, from which detailed molecular structures can be computed. NMR spectroscopy can be applied to molecules both in solution and in the solid state using the magnetic properties of the atomic nuclei. It can provide molecular structure information and is uniquely suited for investigating intermolecular interactions and molecular dynamics in solution in a native‐like environment (Chandra et al., 2022). While there is practically no size limit for target proteins amenable to x‐ray crystallography, NMR investigations of proteins are increasingly difficult with larger molecule size. Most of the more than 14,000 NMR‐based protein structures deposited in the PDB have been reported for proteins below 50 kDa. In special cases, successful structure determination was reported for up to 900 kD (Fiaux et al., 2002); however, using NMR spectroscopy, the dynamic properties and intermolecular interactions can be investigated even in intrinsically disordered proteins or within unstructured regions. Thus, NMR can provide sequence and atom‐specific information on protein–protein and protein–small molecule dynamic interactions in native‐like environments.

This review comprehensively summarizes the current structural knowledge of the major spirochetal outer surface molecules with demonstrated interactions to host ligands (Table 1). Three‐dimensional structures of these key proteins are presented, highlighting their roles in host–pathogen interactions, including adhesion, immune evasion, and tissue colonization. We discuss how structural understanding can guide the design of novel therapeutic strategies, particularly those aimed at blocking critical protein–protein interactions that are essential for the establishment of infection.

**TABLE 1:** Adhesive and immunoregulatory surface‐exposed spirochete proteins and their structures.

Protein and size (kDa)	Host ligand	Experimental structure				
		Method	Analyzed variant and size (kDa)	PDB accession code	Species/strain/serovar	References
**BBK32** (40.8)	Fibronectin, GAGs, C1r	X‐ray	BBK32 CID (16.9)	6N1L	*Bb* s.s. B31	Xie et al. (2019)
		X‐ray	BBK32‐C (16.9) with C1r	7MZT	*Bb* s.s. B31	Garrigues et al. (2021)
		X‐ray	Short BBK32 fragment (1.95) with fibronectin F1 module	4PZ5	*Bb* s.s. B31	Harris et al. (2014)
**DbpA/B** (~21)	Decorin, GAGs	X‐ray	DbpA (18.1)	4ONR	*Bb* s.s. 297	Fortune et al. (2014)
		NMR	DbpA (18.3)	2MTC	*Bb* s.s. N40	Morgan and Wang (2015)
				2LQU	*Bb* s.s. B31	Wang (2012)
		NMR	DbpA (17.9)	2MTD	*B. garinii* PBr	Morgan and Wang (2015)
		NMR	DbpB (18.2)	2MVG	*Bb* s.s. B31	Feng and Wang (2015)
**CspA** (29.2)	Fibronectin, collagen, laminin, plasminogen FH, FHL‐1	X‐ray	CspA (21.4)	4BL4	*Bb* s.s. ZS7	Caesar, Wallich, et al. (2013)
		X‐ray	CspA (21.4)	1 W33	*Bb* s.s. ZS7	Cordes et al. (2005)
**CspZ** (25.5)	Laminin, fibronectin, FH, FHL‐1	X‐ray	CspZ (25–25.5)	7ZJJ	*Bb* s.s. B379	(Marcinkiewicz et al., 2023)
				7ZJK	*Bb* s.s. B408	Marcinkiewicz et al. (2023)
				4BG0	*Bb* s.s. B31	Brangulis et al. (2014)
				4CBE	*Bb* s.s. B31	Brangulis et al. (2014)
		X‐ray	CspZ (25) + FH SCR6‐7	9F7I	*Bb* s.s. B31	Unpublished
				7ZJM	*Bb* s.s. B408	Marcinkiewicz et al. (2023)
**ErpC** (20.3)	Plasminogen, FHR1, 2, 5	X‐ray	ErpC (17.4–18.3)	4BOD, 4BXM	*Bb* s.s. B31	Brangulis et al. (2015)
				4BF3	*Bb* s.s. B31	Caesar, Johnson, et al. (2013)
**ErpP** (20.7)	Plasminogen, FH, FHR1, 2, 5	X‐ray	ErpP (18)	4BOB	*Bb* s.s. B31	Brangulis et al. (2015)
**Fbp—A, B, C** (43)	Fibronectin, C1r	X‐ray	FbpA CID (18)	7RPR	*B. miyamotoi* FR64b	Booth et al. (2022)
		X‐ray	FbpB CID (19.4)	7RPS		
		X‐ray	FbpC CID (18.8)	8EC3	*B. hermsii* HS1	Roy et al. (2023)
**OspA** (29.3)	Plasminogen	X‐ray	OspA (27.6) with murine monoclonal Fab	1OSP	*Bb* s.s. B31	Li et al. (1997)
**OspC** (17.8–22.3)	Plasminogen, fibronectin, DS	X‐ray	OspC (17.7–18.6)	1F1M	*Bb* s.s. HB19	Kumaran et al. (2001)
				1GGQ	*Bb* s.s. B31	Kumaran et al. (2001)
**OspE** (19.2)	Plasminogen, FH	X‐ray	OspE (16.9) + FH 19–20 domains	4 J38	*Bb* s.s. N40	Bhattacharjee et al. (2013)
		NMR	OspE (16.9)	2M4F	*Bb* s.s. N40	Bhattacharjee et al. (2013)
**VlsE** (36)	DS	X‐ray	VlsE (35.1)	1L8W	*Bb* s.s. B31	Eicken et al. (2002)
**TP0751** (25.8)	Laminin, fibronectin, fibrinogen, collagen I + IV	X‐ray	TP0751 (18.1)	5JK2	*T. pallidum* Nichols	Parker et al. (2016)
**FhbB** (11.4)	Plasminogen, factor H	X‐ray	FhbB (10.5)	3R15	*T. denticola* 35405	Miller et al. (2012)
**Msp** (58.3)	Fibronectin	AlphaFold 2	Msp (58.3)	Uniprot Q56256	*T. denticola* 35405	Goetting‐Minesky et al. (2022)
**LigA** (127.9)	Fibrinogen, fibronectin, laminin, collagen I, IV	X‐ray	LigA 8–9 domains (18.8 + 18.9)	8GYR	*L. interrogans*	Kumar et al. (2023)
		NMR	LigA 4 domain (9.5)	2N7S	*L. interrogans* Fiocruz L1‐130	Mei et al. (2015b)
**LigB** (200.8)	Fibronectin, elastin, laminin	NMR	LigBCen2 (10.4)	2MQG	*L. interrogans* Pomona	Mei et al. (2015a)
		NMR	LigB 12 domain (9.6)	2MOG		Ptak et al. (2014)

Abbreviations: *Bb* s.s., *Borrelia burgdorferi* sensu stricto; C1r, complement component C1r; CID, complement inhibitory domain; DS, dermatan sulfate; Fab, fragment of antigen binding region; FH, factor H protein; FHL‐1, factor H like 1 protein; FHR, factor H related protein; GAG, glycosaminoglycan; LigBCen2, part of the 11th and the entire 12th immunoglobulin‐like domain of LigB; TLR2, Toll‐like receptor 2.

## 2 LD AND RF *BORRELIA*

### 2.1 ECM binding molecules

ECM binding molecules in *Borrelia* play a crucial role in the ability of the pathogen to establish infection and persist within the host. These molecules enable the bacterium to interact with various components of the host ECM, facilitating tissue adhesion, immune evasion, and dissemination throughout the body. By binding to ECM structures, *Borrelia* can anchor itself to host cells and tissues, resisting clearance by mechanical and immune defenses (Coburn et al., 2013; Strnad et al., 2015).

#### 2.1.1 BBK32

BBK32 is a fibronectin‐ and GAG‐binding (GAG—glycosaminoglycan) protein that contributes to the infectivity of *Bb* (Fischer et al., 2006; Probert & Johnson, 1998). This protein differs significantly from the most extensively studied fibronectin‐binding proteins: FNBPA from *Staphylococcus aureus* and SfbI from *Streptococcus pyogenes* (Atkin et al., 2010; Schwarz‐Linek et al., 2006). Unlike the multiple fibronectin‐binding repeats (FNBRs) found in FNBPA and SfbI, the fibronectin‐binding sequence of BBK32 is non‐repetitive. FNBRs interact sequentially with fibronectin modules via a tandem zipper mechanism, where the *β*‐strand of FNBR aligns with the C‐terminal strand of the fibronectin module. BBK32 contains a C‐terminal globular domain and an intrinsically disordered N‐terminal domain. The N‐terminal domain of BBK32 includes non‐overlapping binding sites for both host GAGs and fibronectin. The intrinsically disordered portion of the N‐terminal region (residues 21–205) binds to a tandem of fibronectin modules (Probert et al., 2001).

**FIGURE 1:**
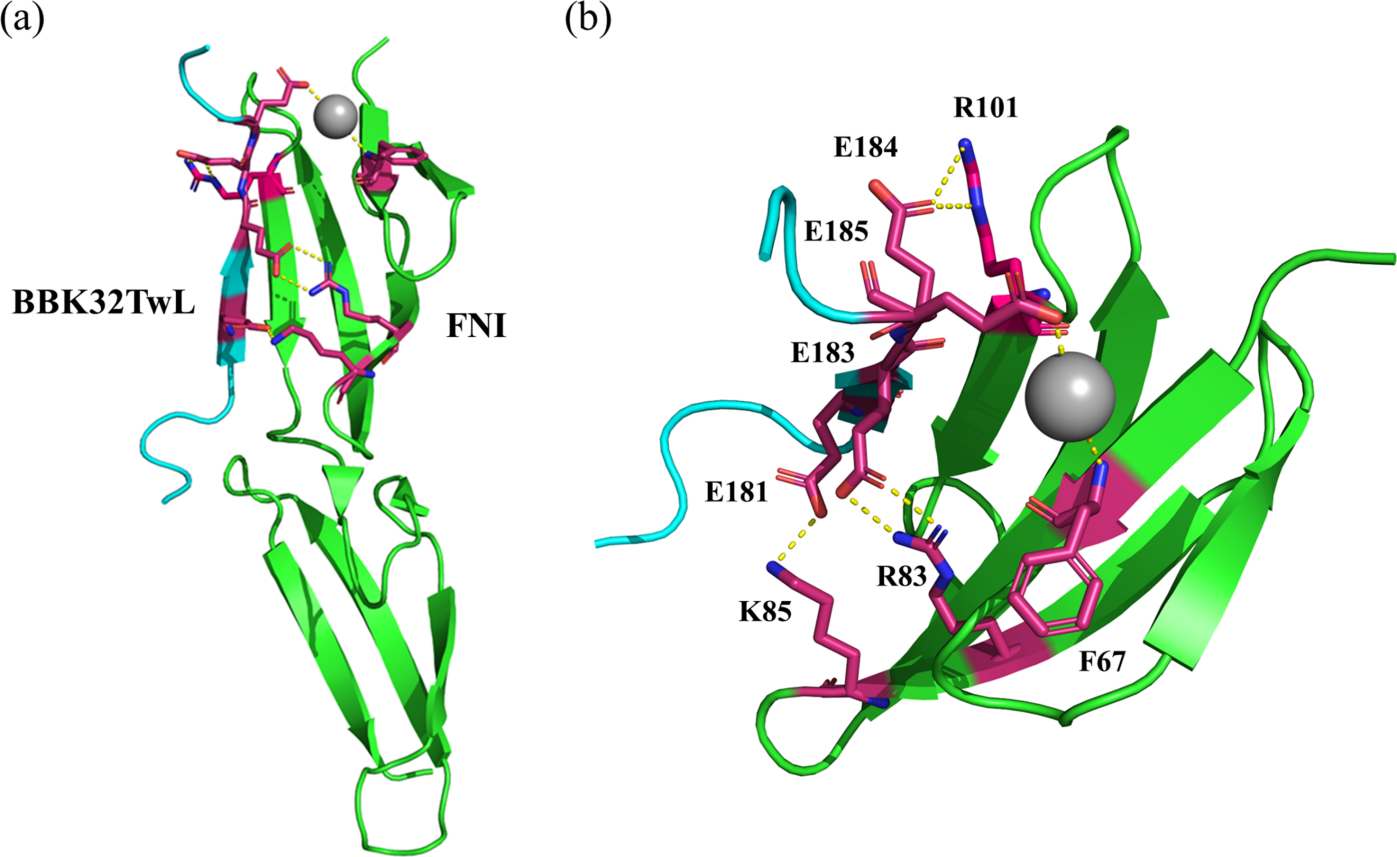
Interactions of BBK32 with fibronectin. Key residues involved in the interaction are depicted as stick models (magenta). (a) Ribbon model of the complete complex structure of the second and third fibronectin modules (FNI, green) in complex with a fragment of BBK32 (BBK32TwL, cyan) from *Borrelia burgdorferi* s.s. strain B31 (PDB code: 4PZ5, resolution 1.96 Å). (b) Detailed view of the BBK32TwL–FNI interaction interface. The ordered water molecule at the interface is represented by a gray sphere. Yellow dotted lines in the picture mark intermolecular interactions E181‐K85 and salt bridges connecting residue pair E183‐R83 and pair E184‐R101. The interaction of E185‐H_2_O‐F67 includes this ordered water molecule and the F67 backbone nitrogen atom.

The sequence of AA residues 120–205 in BBK32 contains a motif (LSGESGEL) similar to that identified in the interaction region of SfbI (LAGESGET). In earlier studies, the primary fibronectin‐binding region in BBK32 was associated with a 32 AA peptide located in the unstructured domain, which shares sequence homology with SfbI (Kim et al., 2004; Probert et al., 2001). In cocci, FNBPA and SfbI are anchored to the bacterial surface via the C‐terminus, unlike in *Borrelia*, where BBK32 is anchored via the N‐terminus (Kim et al., 2004). Further experiments including x‐ray crystallography and NMR spectroscopy revealed several new insights into the fibronectin binding mechanism of BBK32 from *Bb* s.s. (Harris et al., 2014). The adhesin interacts with the N‐terminal region of fibronectin, specifically the 70 kDa N‐terminal region (FN70K fragment). Measurements showed that BBK32 has a higher affinity for FN70K than for intact fibronectin and that the gelatin‐ and fibrin‐binding domains of FN70K are involved in this interaction. Structural analysis of the BBK32‐fibronectin interaction was conducted using various fragments of the fibronectin FNI module, a subunit of the fibronectin monomer (Figure 1a). The interactions were studied using BBK32 fragments, which showed that the FNI module maintains a folded conformation with two‐ and three‐stranded *β*‐sheets (Figure 1a, b).

A 16‐residue fragment of BBK32 (BBK32TwL) interacts with the FNI module fragment in an anti‐parallel orientation, forming hydrogen bonds between the strands (Figure 1b). The glutamate residue side chains in the BBK32TwL fragment play a critical role in the interaction: E183 and E184 form salt bridges with R83 and R101 from the FNI module, respectively. The E185 side chain interacts with F67 through an ordered water molecule (Harris et al., 2014). These interactions are similar to those observed in fibronectin‐binding proteins from *S. aureus* and *S. pyogenes*. An additional interaction unique to BBK32 occurs between E181 and K85 in the FNI module (Harris et al., 2014).

RF spirochetes encode three BBK32 orthologs, fibronectin binding proteins (Fbp), with sequence variations compared to BBK32. The highest sequence identity, between 56% and 62%, was observed for FbpA, while the identity for FbpB ranges from 25% to 30%, and for FbpC, it varies between 22% and 27% (Booth et al., 2022). Surface plasmon resonance showed that only FbpA retains BBK32‐like fibronectin binding activity. The FbpB sequence lacks the motif necessary for interaction with fibronectin, as observed in BBK32 (Booth et al., 2022). Conserved residues involved in the interaction of complement C1r with BBK32‐C were identified in both complement inhibitory domains of the FbpA and FbpB variants (FbpA‐C and FbpB‐C), whereas the conserved lysine residue involved in fibronectin binding was described only in FbpA‐C (Booth et al., 2022).

#### 2.1.2 Decorin binding proteins

**FIGURE 2:**
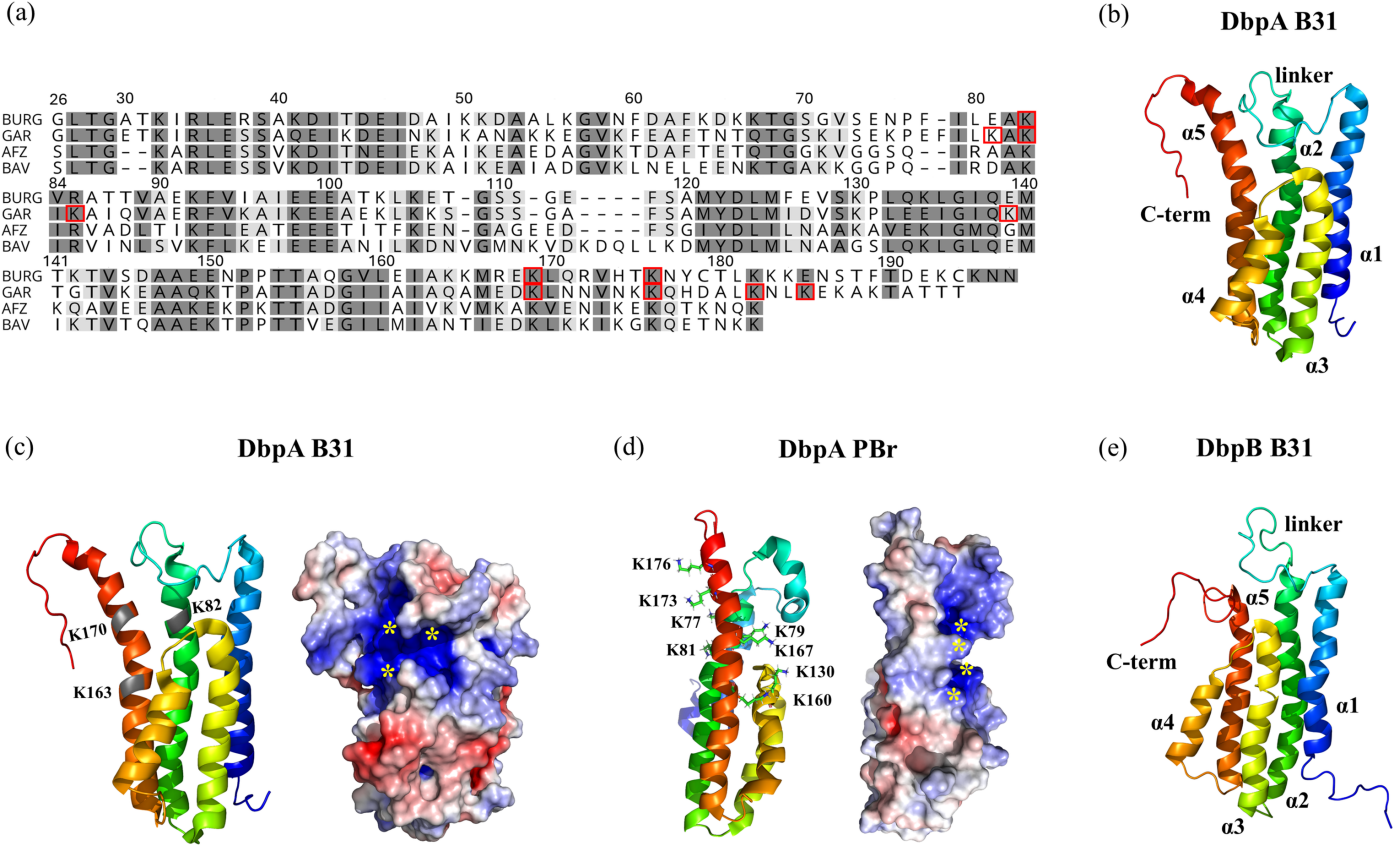
Decorin binding proteins from *Borrelia burgdorferi*. (a) Sequence alignment of DbpA from human pathogenic *B. burgdorferi* sensu lato genospecies, including *B. burgdorferi* sensu stricto strain B31 (BURG), *B. garinii* PBr (GAR), *B. afzelii* A91 (AFZ), and *B. bavariensis* PBi (BAV). (b) Secondary structure layout of DbpA from *B. burgdorferi* B31 (PDB: 2LQU). Solution structures of DbpA from (c) *B. burgdorferi* B31 (PDB: 2LQU) and (d) *B. garinii* PBr (PDB: 2MTD), determined by NMR spectroscopy. The structures are represented as cartoon models with a spectrum‐based color gradient from blue at the N‐terminus to red at the C‐terminus. Electrostatic potentials, calculated using the advanced Poisson–Boltzmann Solver PyMOL plugin, are mapped onto a surface model with a color scale ranging from negative (red) to positive (blue) electrostatic potential values between −5 and + 5 kBT·e^−1^. Conserved lysine residues in the canonical binding site of *Bb* s.s. (K82, K163, and K170) and *B. garinii* (K79, K160, and K167), along with K130 identified by PRE perturbations, are labeled on the ribbon models. In *B. garinii*, a secondary binding site involving residues K77, K81, K173, and K176 is also indicated. All these lysines are marked with red squares in panel (a), and the residue numbering follows that used in the original publications (Morgan & Wang, 2015; Wang, 2012). The corresponding residues in the main binding sites of both variants are marked with yellow stars in the electrostatic potential model. (e) NMR structure of DbpB from *B. burgdorferi* B31 (PDB: 2MVG) with a longer unstructured C‐terminus and shorter *α*5 helix than those of DbpAs.

Decorin binding proteins are surface‐exposed adhesins of *Bb* s.l. that interact mainly with the connective tissue proteoglycan decorin (Guo et al., 1995). Different GAG chains attached to decorin can serve as binding ligands for Dbps. Two homologous forms of decorin binding proteins, DbpA and DbpB, are essential virulence factors crucial for the initial phase of mammalian infection and robust dissemination (Imai et al., 2013; Strnad et al., 2021). The sequence identity between DbpA and DbpB is approximately 40%. DbpB is conserved, while DbpA exhibits sequence variability of up to 42% across different *Bb* s.l. species (Figure 2a). Even relatively minor changes in the Dbp sequence and structure lead to significant differences in ligand binding selectivity and tissue tropism (Lin et al., 2014). This significantly enhances the complexity of LD spirochetes and contributes to the variability of clinical manifestations associated with different species (Strnad & Rego, 2020).

Despite the sequence variability (Figure 2a), the secondary and tertiary structures of DbpA from various *Bb* s.l. species remain quite similar, featuring a bundle of five *α*‐helices and two larger flexible and sequence‐variable regions in the arrangement *α*1‐linker‐*α*2‐*α*3‐loop‐*α*4‐*α*5‐DR (DR = disordered region) (Figure 2b) (Wang, 2012). Helices *α*1, *α*2, and *α*5 are longer than helices *α*3 and *α*4, and the helical bundle is stabilized by a hydrophobic core. The GAG‐binding site in DbpA is formed by alkaline AA located within the long helices (Figure 2c, d). The two variable regions—a dynamic, mostly unstructured linker region between helices *α*1 and *α*2, and the disordered C‐terminal region—are spatially close to the binding pocket (Wang, 2012). Additionally, the loop between *α*3 and *α*4 faces the linker, forming a spatial border of the binding pocket (Figure 2b). The dynamic nature of these regions is reflected by high B‐factors in available x‐ray structures (Fortune et al., 2014). Solution NMR experiments further corroborate this flexibility, with both disordered regions involved in GAG binding (Morgan & Wang, 2013). Notably, the flexible linker in DbpA serves as a negative regulator of GAG affinity (Morgan et al., 2015). Despite the conserved basic AA residues (Lys, Arg) in the binding pocket, DbpA from different species/strains varies in GAG‐binding affinities (Benoit et al., 2011; Fortune et al., 2014). The absence of several basic residues in the linker of DbpA from the *B. garinii* PBr strain results in the highest GAG affinity among tested strains (Benoit et al., 2011). Additionally, linker length influences GAG affinity without altering the basic residues; shorter linkers reduce obstruction in the binding pocket, leading to increased GAG binding (Morgan et al., 2015). For example, DbpA from the *Bb* s.s. N40 strain features basic residues that occupy only the major binding site, resulting in the weakest affinity among the DbpA variants due to linker obstruction. In contrast, the linker in the B31 strain has an additional cluster of basic AAs (the BXBB motif), which apparently compensates for the reduced spatial accessibility of the binding pocket. The C‐terminus of DbpA from the PBr strain lacks the disulfide bond that connects the C‐terminus to helix *α*5, a feature present in DbpA from the B31 and N40 *Bb* s.s. strains (Morgan & Wang, 2015).

GAG‐protein interactions are primarily mediated by electrostatic interactions between the anionic groups (carboxylate and sulfate) of saccharide units and the positively charged side chains of basic AAs exposed on the protein surface (Raman et al., 2005). Electrostatic potential mapping has been used to define the basic binding pocket of DbpA to highlight differences between genospecies and strains (Morgan & Wang, 2015). This basic pocket contains three lysine residues essential for GAG interactions: K82, K163, and K170 in *Bb* s.s. strain B31 (Figure 2c) (Brown et al., 1999). Similarly, *B. garinii* PBr contains these three conserved residues at positions analogous to those in *Bb* s.s. strains (K79, K160, and K167 in PBr) (Figure 2d). In addition to these conserved residues present at the main binding site, electrostatic potential calculations and solution NMR indicate a secondary binding site located at the opposite side of the protein, which includes K77, K81, K173, and K176 (Figure 2d) (Morgan & Wang, 2015).

*B. garinii* PBr DbpA exhibits the highest affinity for both decorin and dermatan sulfate, followed by the B31 strain of *Bb* s.s., while the N40 DbpA shows the weakest interaction affinity (Benoit et al., 2011; Morgan & Wang, 2015). Subsequent experiments focused on the interaction of DbpA variants with short GAG fragments. While hexameric dermatan sulfate had a very low affinity, further NMR titration experiments with heparin oligosaccharide fragments revealed that PBr DbpA has a higher affinity than N40 DbpA, the latter requiring significantly more ligand to induce similar chemical shift perturbations. The largest perturbations are mapped to the linker, helix *α*2, and C‐terminal regions—all close to the basic residues of the proposed GAG‐binding pocket (Morgan & Wang, 2015). For PBr DbpA, the residues with the largest perturbations are located in helix *α*2, the loop between *α*3 and *α*4, and the C‐terminus. Notably, none of the residues in the flexible linker region between *α*1 and *α*2 exhibit large perturbations. ^15^N spin relaxation and heteronuclear Overhauser effect experiments reveal no significant alterations upon GAG binding, indicating that no observable changes in the protein secondary structure or dynamics occur (Morgan & Wang, 2015). Paramagnetic NMR relaxation enhancement (PRE) experiments using a TEMPO‐labeled heparin fragment provided clues regarding the location of the binding site relative to the labeled ligand. TEMPO (2,2,6,6‐tetramethylpiperidin‐1‐oxyl) is a stable aminoxyl radical used in NMR spectroscopy as a spin label, that is, host of a paramagnetic unpaired electron enhancing relaxation in its vicinity. In the case of N40 DbpA, no significant PREs were observed, whereas a notable PRE effect was observed for PBr DbpA in residues I45, K46, A49, E73, and K79, located in helices *α*1 and *α*2 (Morgan & Wang, 2015).

The overall topology of DbpB (Figure 2e) is similar to that of DbpA (Figure 2b), consisting of five *α*‐helices and two unstructured segments, but with several distinct differences (Feng & Wang, 2015). The C‐terminal helix *α*5 in DbpB is shorter, resulting in a longer, disordered C‐terminal tail that is not stabilized by a disulfide bond. This tail also contains more lysine residues than DbpA, significantly enhancing GAG binding in DbpB (Feng & Wang, 2015). Compared to all studied DbpA variants, the canonical GAG‐binding site formed by residues in helices *α*2 and *α*5 of DbpB appears to be less critical. The number of basic residues on this site is half that in DbpA, and the linker BXBB basic motif is absent in DbpB (Feng & Wang, 2015; Morgan & Wang, 2015).

#### 2.1.3 OspA

OspA is an abundant 270 AA lipoprotein found on the surface of *Bb* s.l. spirochetes during laboratory cultivation in artificial media and during colonization of tick midguts. In contrast to the other adhesins mentioned in this review, OspA acts primarily as a tick midgut adhesin (Pal et al., 2000). OspA expression is repressed as *Bb* migrates to the tick salivary glands for transmission to vertebrates (Schwan et al., 1995). OspA so far has been the most targeted antigen in vaccine development for LD (Comstedt et al., 2017; Kamp et al., 2020; Strnad et al., 2020).

**FIGURE 3:**
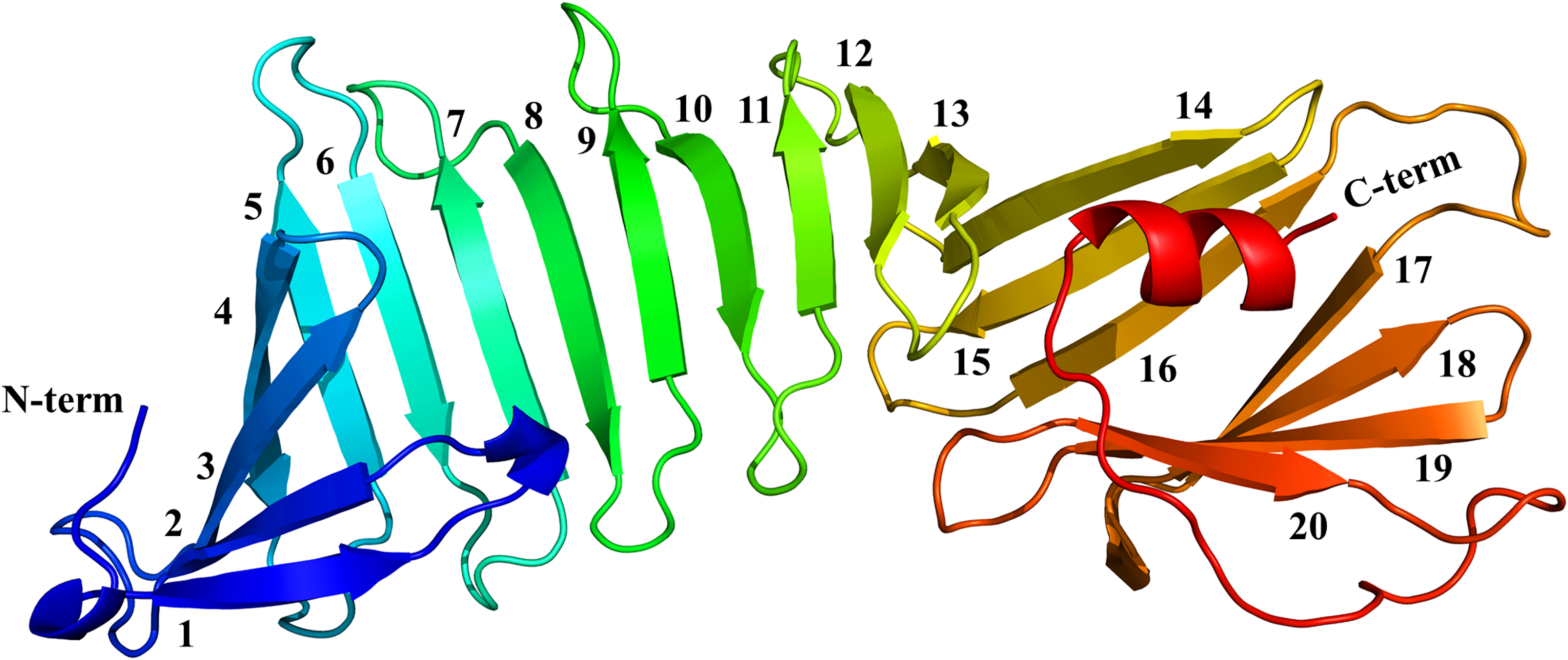
Crystal structure of OspA from *Borrelia burgdorferi* s.s. B31 (PDB: 1OSP, resolution 1.95 Å). The structure is depicted without the murine monoclonal antibody Fab as a cartoon model in spectrum‐style colors ranging from N‐terminus (blue) to C‐terminus (red). *β*‐strands are numbered sequentially from the N‐terminus.

OspA contains 20 *β*‐strands arranged in an anti‐parallel orientation linked by short loops. A short *α*‐helix is located at the C‐terminus. The crystal structure of OspA from *Bb* s.s. strain B31 was obtained from its complex with a Fab fragment of a monoclonal antibody (PDB: 1OSP), resolved at 1.9 Å resolution. The structure of OspA without the Fab is shown in Figure 3 (Li et al., 1997). OspA adopts an unusual dumbbell‐shaped structure consisting of two globular regions connected by a central single‐layer *β*‐sheet (SLB). The first four *β*‐strands at the N‐terminus are oriented perpendicular to the strands of the central SLB, forming a sandwich domain. The two C‐terminal *β*‐sheets formed by strands 14–16 and 17–20 fold with the terminal *α*‐helix into a barrel‐shaped domain that interfaces with the last five strands of the central SLB. Remarkably, both sides of strands 8–10 of the central *β*‐sheet are exposed to the solvent (Li et al., 1997).

OspA has an exceptionally high content of lysine and glutamate residues (25% of all residues), located primarily on the protein surface. The Lys + Glu abundance in OspA exceeds the threshold for entropy reduction defined by Derewenda (2004). These residues have been described as an entropy shield that hinders the crystallization of OspA alone, as some must be immobilized to form a crystal lattice (Makabe et al., 2006). The high abundance of residues with long polar side chains increases the thickness of the central SLB (Pham et al., 1998). Mutational studies of OspA, particularly focusing on the central SLB, revealed a unique hydration pattern, where water molecules are integral to the *β*‐sheet structure and are crucial in keeping the SLB rigid. Replacing the polar residues Lys and Glu with Ala and Ser in the central SLB reduces the protein solubility but facilitates its crystallization (Makabe et al., 2006).

NMR studies on OspA have complemented the x‐ray crystallography data by providing additional insights into the protein conformation and dynamics (Pham & Koide, 1998). OspA is highly rigid and features a structurally unique central *β*‐sheet that lacks a hydrophobic core (Pham & Koide, 1998), in contrast to most anti‐parallel *β*‐sheets in proteins, which are typically amphipathic, with one side forming a hydrophobic core (Tsutsumi & Otaki, 2011). A large rotational diffusion anisotropy of the molecule was found by nuclear spin relaxation (Pham & Koide, 1998). The central SLB lacks the characteristic movements associated with *β*‐sheets, such as bending and stretching (Pham & Koide, 1998). The “sandwich‐like” shape of the central SLB, with its attached globular ends, reveals the burial of non‐polar surfaces at a similar level to that found in small globular proteins (Li et al., 1997; Pham & Koide, 1998).

#### 2.1.4 VlsE

VlsE is an outer surface exposed protein enabling *Bb* s.l. to avoid the acquired immunity of the host (Lawrenz et al., 2004). A key feature of the immune evasion strategy of *Bb* is recombination at the *vls* locus. During mammalian infection, the *vls* locus undergoes continuous segmental recombination, generating a large number of VlsE variants (Zhang et al., 1997). The *vls* locus contains an expression site (*vlsE*) and an array of approximately 15 silent *vls* cassettes that share more than 90% nucleotide sequence identity. The expression site consists of a leader sequence, an N‐terminal constant region, a variable cassette region flanked by short direct repeats on both sides, and a constant C‐terminal domain (Zhang et al., 1997). Both silent cassettes are approximately 500 base pairs long. The *vlsE* expression sequence and the silent cassettes are separated by a short intergenic region containing an inverted repeat sequence capable of forming a stable stem‐loop structure (Hudson et al., 2001).

**FIGURE 4:**
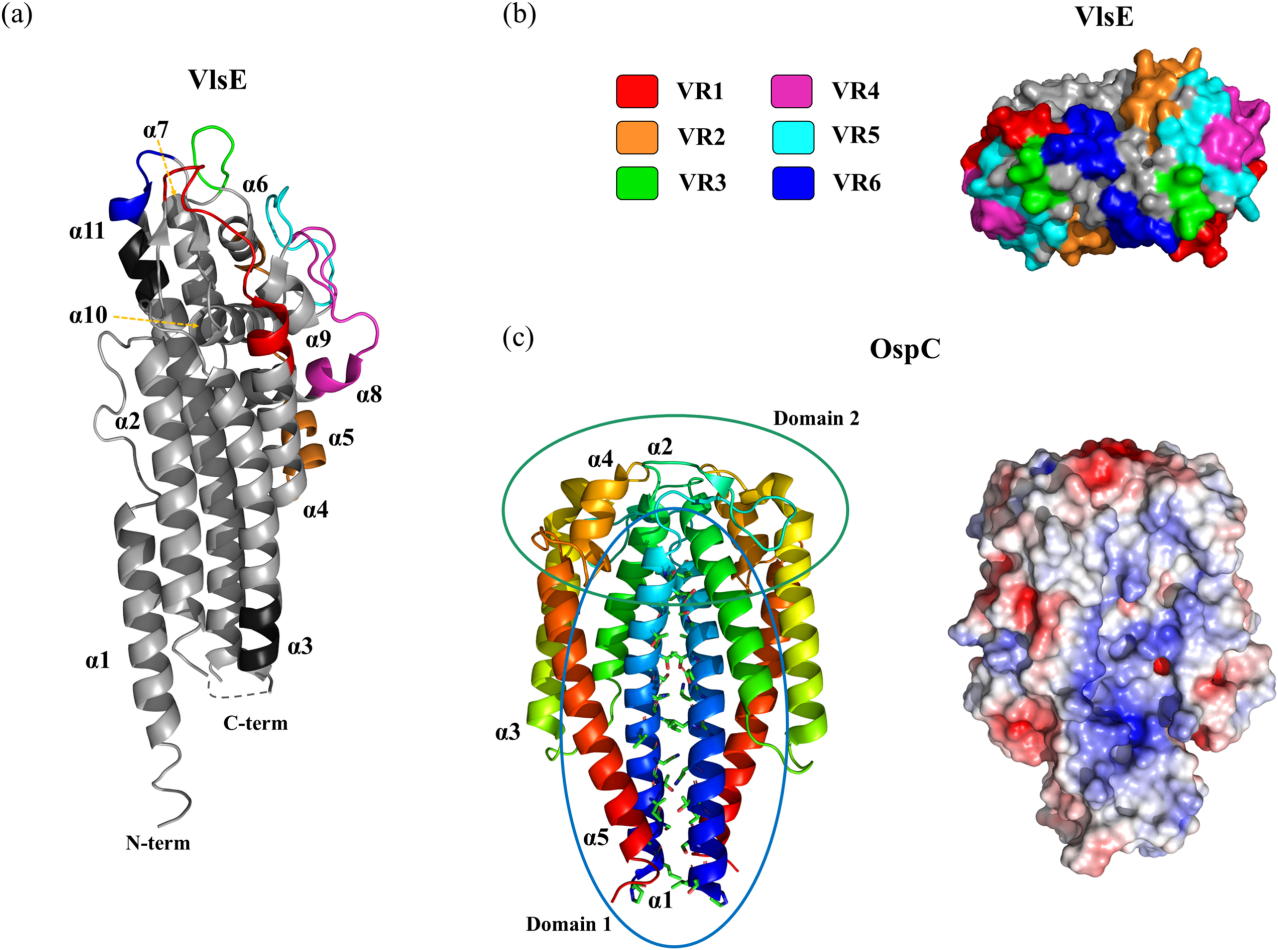
Structure of VlsE and OspC. (a) X‐ray crystal structure of the VlsE monomer from *Borrelia. burgdorferi* s.s. B31 (PDB: 1L8W). The variable regions are distinctly colored and separated from the constant regions by dark gray segments. The dotted line indicates weak electron density in the experimental x‐ray structure. (b) Structural model showing the Van der Waals surface of the proximal part of the VlsE dimer, with variable regions (VR1‐6) highlighted in different colors. (c) X‐ray crystal structure of the OspC dimer from *B. burdorferi* s.s. strain HB19 (PDB: 1F1M, resolution 1.8 Å). Each monomeric unit is color‐coded from N‐terminus (blue) to C‐terminus (red) using a spectrum‐based scheme. Two main ligand binding domains are marked with ellipses. The interacting residues forming the dimeric interface are indicated by stick models. The negative electrostatic potential surface of OspC is marked in red and the positive potential in blue, with a scale ranging from −5 to 5 k_B_T.e^−1^.

The structure of VlsE was determined from crystals containing four protein molecules in the asymmetric unit. The absence of a hydrophobic patch at the molecular interface, combined with thermal denaturation analysis via differential scanning calorimetry and far‐UV circular dichroism, suggests that native VlsE exists as a dimer on the *Bb* surface (Jones et al., 2001). A monomeric VlsE molecule contains 11 *α*‐helices and four short *β*‐strands with three residues each (Figure 4a). The electron density map shows that the first 16 residues of the N‐terminus and the last 12 residues of the C‐terminus are disordered. Helices *α*1, *α*2, and *α*11 form the core of the conserved regions of VlsE. The variable cassette region begins with the direct repeat sequence located near the membrane‐proximal part of the *α*3 helix (Figure 4b). Together, *α*1, *α*2, *α*3, and *α*11 form the proximal region of VlsE. The loop connecting *α*2 and *α*3 is disordered. Helices *α*4 to *α*10 comprise the core of the distal region, which also contains all four *β*‐strands. Loops in the distal region cover the helices. Compared to the variable regions, the invariant regions of VlsE primarily form secondary structures with minimal surface exposure, comprising approximately 60% of the cassette region (Eicken et al., 2002).

#### 2.1.5 OspC

OspC is a highly variable protein of LD *Borrelia* and is coded by one of the most polymorphic genes in the *Bb* genome (Jauris‐Heipke et al., 1993; Wang et al., 1999). OspC is upregulated during tick feeding (Schwan et al., 1995). It remains expressed in the early phases of mammalian infection but is subsequently downregulated (Tilly et al., 2006). An OspC mutant lacking the ability to bind either dermatan sulfate or fibronectin shows severely impaired tissue colonization, with tissue levels comparable to those of an OspC‐deficient strain (Lin et al., 2020). While the genetic variability within one allelic group is less than 1%, sequence variability between allelic groups can be as high as 45% (Theisen et al., 1993). Despite this high degree of genetic variability, OspC is structurally conserved.

Crystallized OspC (PDB: 1F1M; *Bb* s.s. strain HB19) forms an almost entirely helical homodimer, with its axis aligned parallel to the long helices that create the dimer interface, which accounts for more than 30% of the total protein surface area (Figure 4c). Each OspC monomer consists of four long *α*‐helices (*α*1, *α*2, *α*3, and *α*5) arranged in an anti‐parallel fashion, forming the protein core, along with a short helix *α*4 and two short *β*‐strands. The two anti‐parallel *β*‐strands extend toward the upper part of the second monomeric unit. Dimerization of OspC occurs primarily through interactions between *α*1 helix of each monomer in a parallel orientation stabilized by hydrophobic contacts. The protein contains two solvent‐accessible regions referred to as ligand‐binding domains 1 and 2. The highly conserved domain 1 is located at the dimer interface, where helix *α*1 from each monomeric unit comes together, exhibiting a positive electrostatic surface potential. In contrast, the more variable domain 2 is positioned at the top of the dimer and is characterized by a negative electrostatic surface potential (Figure 4c) (Kumaran et al., 2001).

The conserved structure is reflected in the AAs of the variable domain 1. Three conserved residues, K60, E61, and E63, project their side chains from the hydrophobic interface into the solvent‐accessible pocket of domain 1 (Kumaran et al., 2001). However, only E61 has been described as critical for the infection‐related function of OspC in mammals (Earnhart et al., 2010). Mutation assays, including E61Q and E63Q, showed a higher negative charge in domain 1, resulting in increased plasminogen binding (Earnhart et al., 2010) compared to wild‐type OspC. Additionally, lysine residues located in the *α*3 helix have been proposed to be critical for fibronectin‐ and dermatan sulfate‐binding activity (Lin et al., 2020), highlighting the importance of electrostatic interactions in ligand recognition. While multiple host ligands have been identified by ELISA and surface plasmon resonance to bind OspC (Caine et al., 2017; Lin et al., 2020), the specific domains or sites responsible for these interactions have yet to be fully defined by structural tools.

Comparing OspC with VlsE reveals several structural similarities. Both proteins have the ability—at least the propensity, in the case of VlsE—to form dimers under native conditions. Shared features include the structural proximity of their N‐ and C‐termini and the presence of long helices near the membrane‐proximal region, which form the dimer interface (Eicken et al., 2002; Kumaran et al., 2001).

### 2.2 Structural insights into the binding of immunoregulatory molecules

Besides mediating attachment to ECM components and cell surfaces, multiple surface‐exposed molecules of *Borrelia* interact with the host immune system (Strnad et al., 2023). For a subset of *Borrelia* immune evasion proteins, high‐resolution structural data has elucidated their molecular interactions—particularly with components of the complement system. The complement system is an evolutionarily conserved, crucial mechanism of innate immunity, consisting of a complex of proteins that are activated in a cascade. The first component of the classical pathway, the C1 protein complex, is composed of six C1q, two C1r, and two C1s units. C1q has a globular head and a long collagen‐like tail (Reid & Porter, 1976). The six C1q units are linked together by their tails and symmetrically surrounded by two C1r and two C1s units (Reid, 2018). Binding C1q to a pathogen leads to activation of C1r, which subsequently activates C1s. C1s acts as a serine protease, triggering the next steps of the activation pathway (Gaboriaud et al., 2004).

#### 2.2.1 Complement C1r and BBK32

**FIGURE 5:**
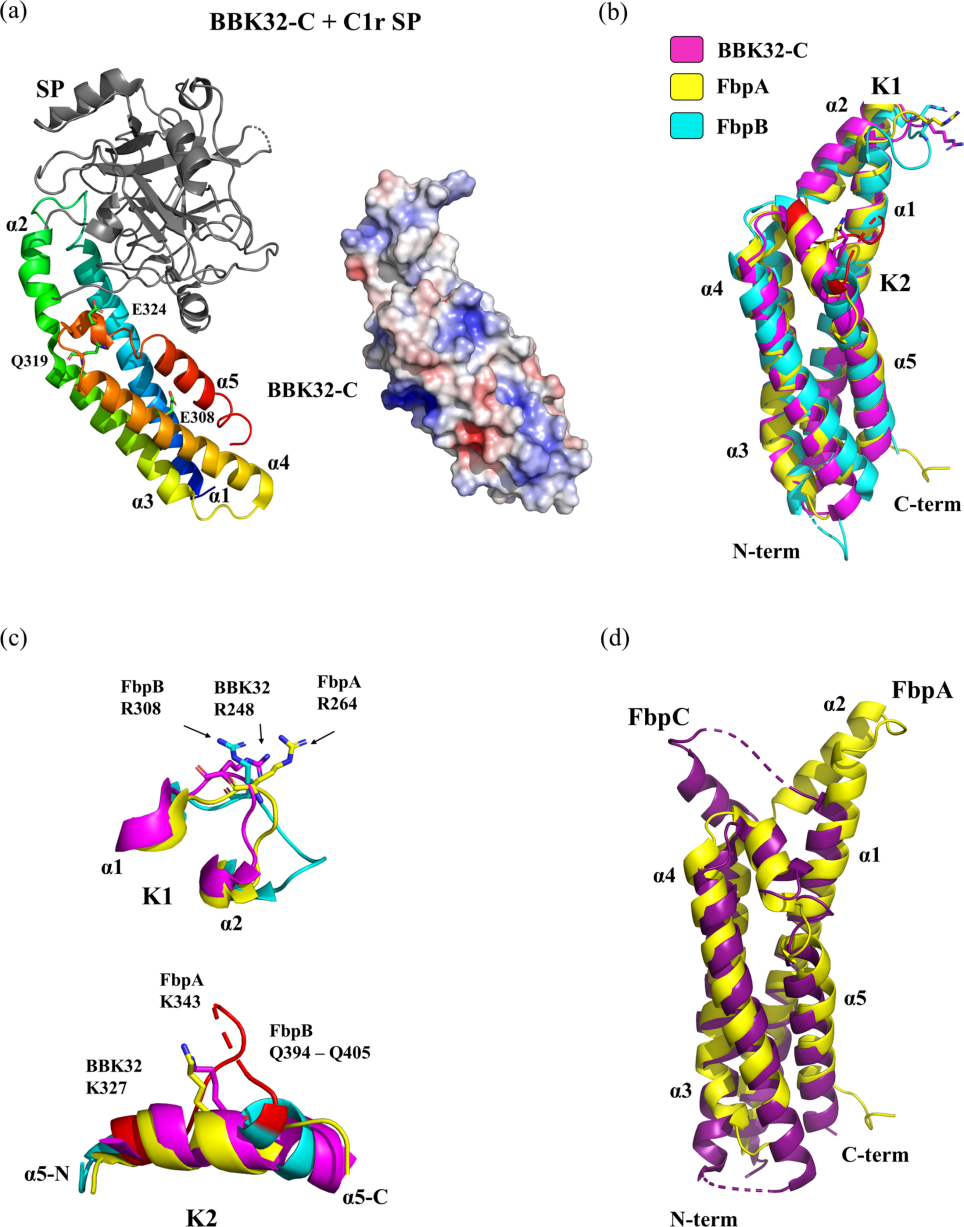
Complement regulatory BBK32 orthologs of *Borrelia*. (a) Crystal structure of the complex formed by the C‐terminal globular domain of BBK32‐C (residues 210–347) from *Borrelia burgdorferi* s.s. B31 interacting with the complement autolytic fragment of C1r (residues ~300–705) is presented; (PDB: 7MZT, resolution 4.1 Å). BBK32‐C is color‐coded using a spectrum, with the N‐terminus in blue and the C‐terminus in red. The serine protease (SP) subunit of C1r is highlighted in gray. The electrostatic potentials of BBK‐32C are mapped onto a surface model using a color scale ranging from negative (red) to positive (blue). (b) Structural comparison of complement inhibitory domains of BBK32 from LD *Borrelia* with Fbp structures from RF *Borrelia*. BBK32‐C from *Bb* s.s. is shown in magenta (PDB: 6N1L, resolution 1.72 Å), FbpA‐C from *B. miyamotoi* in yellow (PDB: 7RPR, resolution 1.9 Å), and FbpB‐Δ5C from *B. miyamotoi* in cyan (PDB: 7RPS, resolution 2.09 Å). The structural alteration of FbpB‐Δ5C, caused by the inserted sequence Q394–Q405, is marked in red. Conserved residues at the K1 and K2 sites are depicted as stick models. (c) Detailed view of the K1 site, located in the loop between helices *α*1 and *α*2, and the K2 site, surrounding the functionally critical residue K327. Conserved arginine residues at the K1 site are shown as stick representation: BBK32‐C in magenta, FbpA‐C in yellow, FbpB‐Δ5C in cyan (inserted sequence Q394–Q405 in red). An additional lysine residue, K343, in FbpA‐C is exposed in the K2 site due to the extended helical structure. Weak electron density for residues 401–403 in the diffraction analysis is indicated by a dashed line. (d) Structure comparison of FbpC‐C from *B. hermsii* (PDB: 8EC3, resolution 1.5 Å) in deep purple, with the structure of FbpA from *B. miyamotoi* in yellow.

The C‐terminal globular part of BBK32 (BBK32‐C) binds specifically to C1r as identified by surface plasmon resonance (Garcia et al., 2016). The structure of nearly complete *Bb* s.s. strain B31 BBK32‐C was determined by x‐ray crystallography (PDB: 6N1L, resolution 1.7 Å) (Xie et al., 2019). The resolved structure consists of a helical bundle composed of five *α*‐helices (Figure 5a). Four *α*‐helices (1, 3, 4, and 5) form an anti‐parallel core bundle, whereas helix *α*2 protrudes from the bundle at an acute angle. Helix *α*2 is rotated by approximately 120° relative to helix *α*3 and forms hydrophobic contacts with helix *α*1, with alanine residues facing helix *α*1. Electrostatic potential calculation characterized the protein structure as a set of positively charged adjacent regions, with a larger negatively charged region around the point where the N‐terminal and C‐terminal *α*‐helix ends meet (Figure 5a). The areas with the highest positive charge are formed by lysines in helices *α*3 and *α*4 (around K274 and K310) and helix *α*2 (around K254 and K258) (Xie et al., 2019).

*Bb* s.s. BBK32 orthologs have been identified in other *Bb* s.l. genospecies, with approximately 90% sequence identity in *B. afzelii* and around 70% in *B. garinii*. While both orthologs bind purified C1r protease with high affinity, the *B. garinii* ortholog displayed significantly reduced in vitro complement inhibitory activity compared to those from *Bb* s.s. and *B. afzelii* (Xie et al., 2019). A sequence alignment of the C‐terminal part of all three orthologs revealed and identified three variable residues that are non‐conservatively substituted in *B. garinii* compared to the other orthologs. In *B. garinii*, these residues are changed to K308, K319, and Q324, whereas in *Bb* s.s. and *B. afzelii*, the corresponding residues are E308, Q319, and E324, respectively (Figure 5a). Mutation analysis of these three residues highlights their contribution to C1r inhibitory activity. The positions of these residues were predicted, with all three exposed to solvent: residue 308 is located in the middle of helix *α*4, while residues 319 and 324 are situated in the short loop connecting helices *α*4 and *α*5 (Figure 5a) (Xie et al., 2019).

A combination of techniques was used to characterize the structural properties of the complex formed by BBK32‐C interacting with human C1r‐CCP2‐SP (CCP—complement control protein, SP—serine protease) (Garrigues et al., 2021). Despite the lower resolution obtained by x‐ray diffraction at 4.1 Å, a reasonable structure was achieved. The interface area between BBK32‐C and the SP domain (Figure 5a) of C1r was determined to be 1340 Å^2^ of the buried surface area. The reliability was supported by comparison with previously published crystal structures of BBK32‐C (PDB: 6N1L) and the activated form of the C1r fragment, C1r‐CCP2‐SP (PDB: 1MD8, resolution 2.80 Å) (Xie et al., 2019). Interaction analysis predicted 11 BBK32‐C residues (L236, T239, T242, Y245, R248, N251, Y323, E324, E327, T331, R337) from helices *α*1 and *α*5 and the short loop between helices *α*1 and *α*2, which might be involved in binding between BBK32‐C and C1r‐CCP2‐SP (Garrigues et al., 2021).

RF *Borrelia* orthologs of BBK32‐C, proteins FbpA‐C and FbpB‐C, show differences in C1r interactions. FbpA‐C can bind to both forms of C1r, the inactive zymogen and the active form, whereas FbpB‐C selectively binds and inhibits only the active form of C1r (Booth et al., 2022). *B. miyamotoi* FbpA‐C and FbpB‐C share a similar structural pattern with BBK32‐C, but they are divergent in the functionally critical region of the protein (Booth et al., 2022). X‐ray crystallography has resolved the structures of FbpA‐C (PDB: 7RPR, resolution 1.9 Å) and FbpB‐Δ5C (PDB: 7RPS, resolution 2.09 Å), which were overlaid with the BBK32‐C structure (PDB: 6N1L) (Figure 5b). Due to difficulties in obtaining crystals of intact FbpB‐C suitable for further experiments, a short deletion was made at the C‐terminus, designated as FbpB‐Δ5C (Booth et al., 2022).

The structural comparison reveals that all three proteins share a similar fold, characterized by a core four‐helix bundle (helices *α*1, *α*3, *α*4, and *α*5), with helix *α*2 protruding from the core. In all three proteins, residue R248 (in BBK32 nomenclature) is conserved (Figure 5c). It is located within a small loop between helices *α*1 and *α*2 in the K1 site, which is one of two regions critical for interaction with C1r. In contrast, some minor structural alterations occur around the BBK32 residue K327, within the K2 site, the second C1r interaction moiety. The K2 site in FbpA‐C exposes an additional lysine (K343) through an extended turn in helix *α*5 (Figure 5c). The structural alteration of FbpB‐Δ5C in this region is much more significant. Weak electron density of residues 401–403 in the diffraction analysis of FbpB‐Δ5C indicated a loss of secondary structure in helix *α*5 caused by a loop insertion between Q394 and Q405, which is unique compared to the other two proteins. Additionally, the proline residue P397 disrupts the helical structure of FbpB (Booth et al., 2022; Xie et al., 2019).

FbpC‐C, BBK32‐C, and FbpA‐C interact with both zymogen and active forms of human C1r. This interaction inhibits the classical pathway of complement activation, protecting serum‐sensitive *Bb* strains from complement‐mediated killing (Roy et al., 2023). In the crystal structure of FbpC‐C from *B. hermsii*, determined at 1.5 Å resolution (PDB: 8EC3), the folding pattern closely resembles that of other orthologs (Figure 5d). Structure comparison revealed discrepancies in the K1 site among BBK32‐C, FbpA‐C, and FbpB‐C variants. The interhelical angle formed by helices *α*1, *α*2 and *α*3 is 75° in FbpC, while in BBK32, FbpA, and FbpB, it varies between 20° and 25°. A second disturbance in this region is a loss of resolution, manifested by a dispersion of electron density in the positions of residues 247–254. Compared to BBK32 and FbpA and FbpB, the structure around the K2 site demonstrates unwinding of helix *α*5 into a larger loop structure ranging from residue 344 to residue 354 (Booth et al., 2022; Roy et al., 2023).

Molecular dynamics simulations revealed protein conformational changes of C*α* carbons in BBK32‐C, FbpA‐C, FbpB‐C, and FbpC‐C, as indicated by root mean square deviation (RMSD) values in the nanosecond range. While most simulations converged after 100 ns, FbpC‐C required 500 ns. Higher RMSD values for FbpC‐C compared to the others (3.29–3.81 Å vs. FbpA‐C with the highest range of 2.36–3.04 Å) indicate an increase in structural flexibility of the protein. The simulation evaluation of local flexibility by RMSD values for individual residues suggested that the K1 site, as the main interaction site, is dynamic in all four C1r inhibitor proteins. Evidently, the K1 interaction site of FbpC‐C is the most dynamic region within the C1r inhibitor proteins of *Borrelia* (Roy et al., 2023). Even though the dynamics of the K2 interaction site in all C1r inhibitors are less pronounced compared to K1, the K2 site of FbpC‐C is still more dynamic than those of BBK32‐C, FbpA‐C, and FbpB‐C. Cross‐correlation molecular dynamics analysis also indicates that interaction sites K1 and K2 move in an anticorrelated fashion. The simulation results for the K1 site are also in accordance with the lack of electron density in the dynamic loop of the protein crystal structure (Roy et al., 2023).

#### 2.2.2 CRASPs

*Bb* expresses several CRASP that are functionally related but structurally heterogeneous. All these CRASP proteins have been shown to bind to the human complement regulator FH, leading to complement inactivation (Alitalo et al., 2002). CRASP‐1 (CspA) and CRASP‐2 (CspZ) can bind to FH‐like protein 1 (FHL‐1) (Hartmann et al., 2006; Kraiczy et al., 2004). Other proteins, including OspE paralogs, such as CRASP‐3 (ErpP), CRASP‐4 (ErpC), and CRASP‐5 (ErpA), bind to various FH‐related proteins that are sequentially related to FH (Brissette et al., 2008) and have been identified as plasminogen‐binding proteins (Brissette, Haupt, et al., 2009). CspA has been found to bind several human proteins, including fibronectin, laminin, plasminogen, and various types of collagens (Hallström et al., 2010). The binding of CspA to FHL‐1 is stronger than its binding to FH (Kraiczy et al., 2004). CspA polymorphism is associated with differences in FH binding affinity. Sequence alignment of CspA orthologs reveals sequence identities ranging from 45% to 54%, and interactions with FHL‐1 show variations in binding affinity (Wallich et al., 2005). The “regulators of complement activation” gene cluster also encodes five FH‐related proteins (FHRs). Both FH and FHL‐1, along with the FHRs, consist of short consensus repeat domains (McRae et al., 2002). The high structural similarity between the C‐terminal domains of FH and FHRs allows them to bind to similar ligands (Goicoechea de Jorge et al., 2013).

##### CspA

**FIGURE 6:**
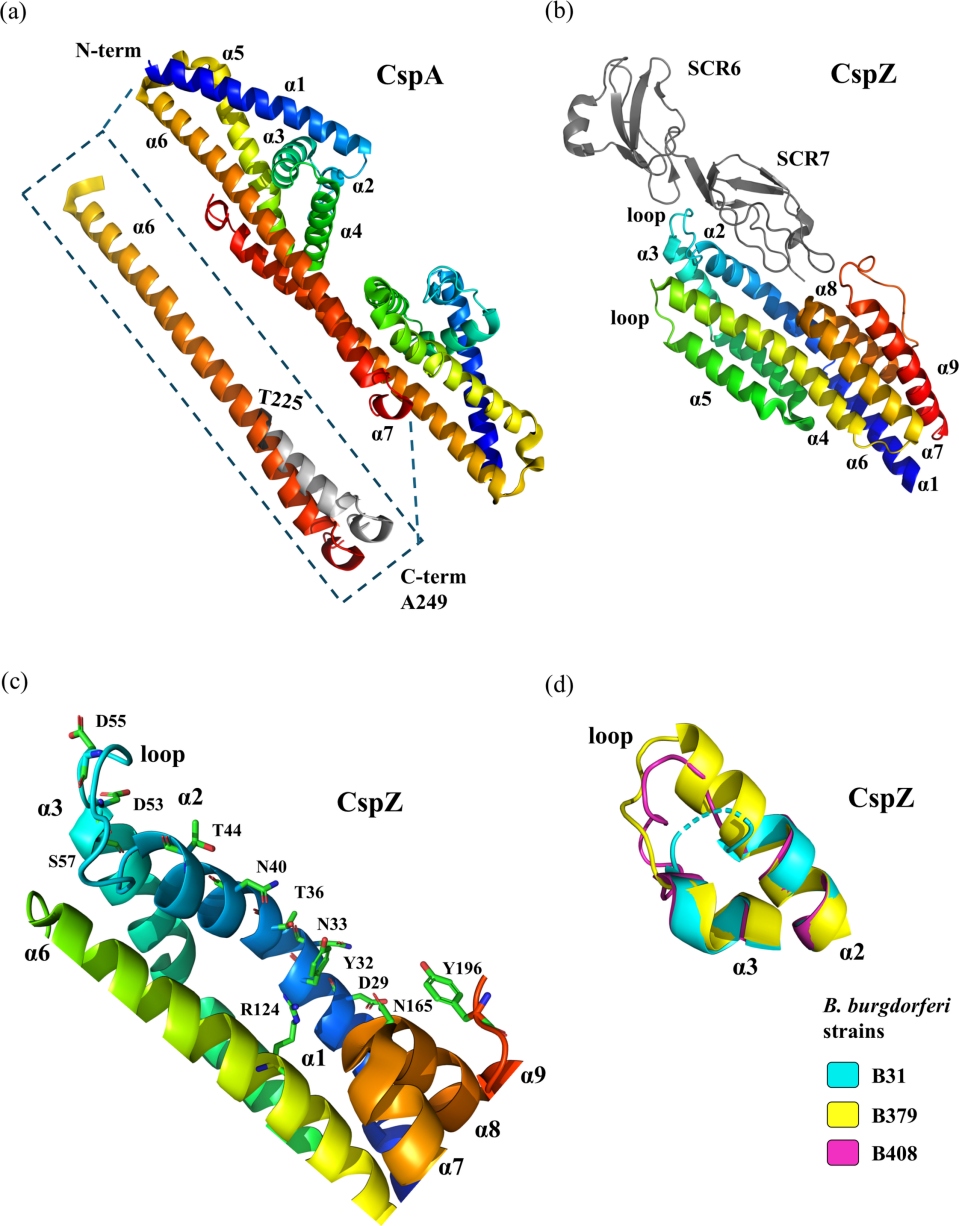
Structure of CRASP proteins CspA and CspZ. (a) X‐ray crystallographic structure of the CspA dimer from *Borrelia burgdorferi* s.s. strain ZS7 (PDB: 4BL4, resolution 4.06 Å), representing 181 residues of the 251 amino acids present in the full‐length mature protein. The cartoon model is shown with a spectrum‐style color gradient from blue (N‐terminus) to red (C‐terminus). The structural difference in the C‐terminal helix of another CspA from *B. burgdorferi* s.s. strain ZS7 (PDB: 1W33), caused by bending between residues T225‐D227, is colored in gray. (b) X‐ray crystal structure of the CspZ complex from *B. burgdorferi* strain B408 with factor H (PDB: 7ZJM, resolution 2.59 Å). The structure of CspZ is shown as a cartoon model with a spectrum‐based color gradient ranging from blue (N‐terminus) to red (C‐terminus). The SCR‐6 and SCR‐7 units of factor H are colored gray. (c) Residues of CspZ involved in the interaction with factor H are labeled and shown as stick models. (d) An overlay of structures from *B. burgdorferi* strains B31 (PDB:4CBE), B379 (PDB:7ZJJ), and B408 (PDB:7ZJK) shows strain‐dependent variability in the CspZ polymorphic loop.

The dimeric crystal structure of CspA (Figure 6a) engages the cleft between the two monomeric units, which is the primary region for interaction with FH (Cordes et al., 2005; Cordes et al., 2006). The C‐terminal part of CspA plays a crucial role in its properties, with the longest helix *α*6 being essential for dimer formation (Cordes et al., 2006). C‐terminal helix *α*7 is also important for FH binding (Cordes et al., 2006; Kraiczy et al., 2009). Multiple forms of CspA structures have been solved by x‐ray diffraction (PDB codes 4BL4 from *Bb* s.s. ZS7 (Caesar, Wallich, et al., 2013), 1W33 from *Bb* s.s. ZS7 (Cordes et al., 2005), 5A2U from *Bb* s.s. B31 [unpublished structure]). The superposition of the dimer from 4BL4, representing 181 residues of the 251 AAs in the full‐length protein (missing residues 27–69), onto the experimental structure 1W33 reveals divergence in helix *α*6 between residues 225 and 227, resulting in a bend of 16.8° in the structure (Figure 6a). These findings suggest some degree of flexibility between the subunits, potentially leading to conformational changes in the dimer. An increase in the angle of curvature of helix *α*6 could open the clutch around FH, while another possibility is the clamping of the dimer onto FH. Despite this flexibility, the conserved C‐terminal dimer‐lock structure suggests that residues 240–250 are necessary for dimer assembly, remaining unaffected by the observed flexibility in the different protein forms (Caesar, Wallich, et al., 2013).

Although dimer or oligomer formation is a common crystallization artifact, CspA dimerization has been specifically described as essential for ligand binding. Mutation studies mapped the dimerization interface and inter‐dimer cleft region and showed that disruption of the CspA dimer led to a complete loss of FH and FHL‐1 binding. The substitution of inter‐dimer cleft localized residues K136R, K141T, and E147K in helix *α*4 reduced the interactions with both ligands, and substitution L146H completely eliminated binding. Surface plasmon resonance revealed that all mentioned mutants of residues E234A, H235A, K238A, Y239A, F243H, D244A, T245A, and the double mutant K241E‐D242A (Figure 6a, in gray region) showed reduced FH and FHL‐1 binding. C‐terminal substitutions Y240A, D242A, and L246D cause a total loss of binding activity (Kraiczy et al., 2009).

##### CspZ

CspZ is a highly conserved protein among LD *Borrelia* species, exhibiting a sequence identity of over 97% among *Bb* s.s. strains, 81.9%–96.9% for *B. garinii*, and 90.8% for *B. afzelii* (Rogers & Marconi, 2007). Besides its interaction with ECM components, such as laminin and fibronectin, CspZ has been identified as an FH‐binding protein (Hallström et al., 2010; Haupt et al., 2007). Variability in CspZ variants has been observed in relation to FH binding, specifically in the SCR 6 and SCR 7 domains of FH. The CspZ binding site contains a polymorphic motif that is thought to contribute to host‐specific FH binding.

Structurally, CspZ is a single‐domain protein consisting of nine *α*‐helices folded around a hydrophobic core (*α*2, *α*4, *α*5, and *α*6), which includes two disordered loops between *α*2–*α*3 and *α*5–*α*6 (Figure 6b). The protein can be described as a two‐lobe molecule: the N‐terminal lobe contains a six‐*α*‐helix bundle (*α*1–*α*6), which has been identified as the FH‐binding region, while the C‐terminal lobe is composed of three *α*‐helices (*α*7–*α*9). Anti‐parallel helices *α*7 and *α*8 form a helix‐turn‐helix motif, and the last helix *α*9 is connected to *α*8 by a long loop (Brangulis et al., 2014).

To explore the polymorphism of CspZ and the FH interaction moiety, the x‐ray structures of the complex between CspZ from several *Bb* s.s. strains (B379, B408, B31) and SCR‐6 and SCR‐7 of FH were determined (Marcinkiewicz et al., 2023). This revealed that the residues in contact with FH are predominantly located in helix *α*2 and the loop between helices *α*2 and *α*3 (Figure 6b), with additional residues found in other regions. Specifically, the residues interacting with SCR‐7 included D29, Y32, N33, T36, N40, and T44 in helix *α*2, R124 in helix *α*6, N165 in helix *α*7, and Y196 in helix *α*9. Residues D53, D55, and S57, which interact with both SCR‐6 and SCR‐7, are located in the loop between helices *α*2 and *α*3 (Figure 6c). Most of these FH binding residues are conserved among CspZ variants, with the exception of N33 and D53. However, mutation studies of these residues did not affect ligand binding, indicating that they do not explain the differences in CspZ‐FH interactions among the variants (Marcinkiewicz et al., 2023). The main area of polymorphism in CspZ is in the C‐terminal part of helix *α*2 and the following loop connecting to helix *α*3 (Figure 6d). The polymorphic region of CspZ from *Bb* strain B379 includes an extension of helix *α*2 created by a unique duplication of residues I42, M43, T44, and Y45. This extension is an obstacle for FH binding, explaining the variability in FH binding among the different CspZ variants (Marcinkiewicz et al., 2023).

#### 2.2.3 OspE and Erp proteins

**FIGURE 7:**
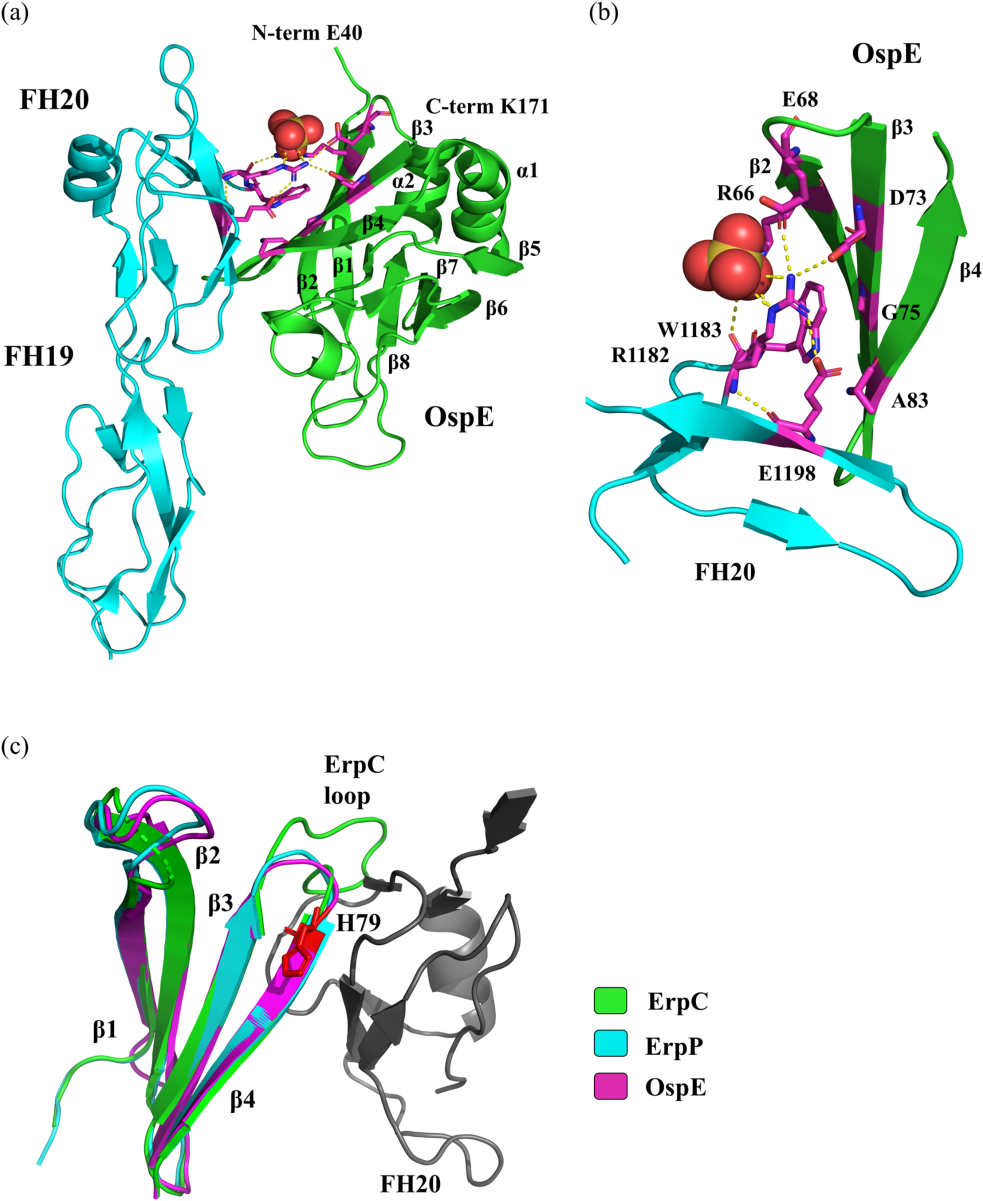
OspE‐factor H complex and comparison with ErpC. (a) X‐ray crystal structure of the OspE backbone from *Bb* s.s. strain N40 (residues 42–171) interacting with the FH19 and FH20 units of factor H (PDB: 4J38). The FH19 and FH20 units of factor H are colored cyan, while the structured backbone of OspE is shown in green. Interacting residues are displayed as a magenta stick model, with a sulfate ion shown as a ball model. (b) Detailed view of the OspE‐FH20 interface, focusing on a segment of OspE (residues D62–F89) in green and FH20 (residues Y1176–R1203) in cyan. The interacting residues are represented as magenta stick models, with the sulfate ion as a ball model. Interactions calculated using PyMOL are indicated by yellow dashed lines. Key interactions stabilizing the complex include an ion triplet formed by R1182 of FH20 with E68 and D73 of OspE, as well as the interaction between R1182 and the sulfate ion, and R66. Additionally, R1182 forms an intramolecular bond with E1198. (c) Alignment of the structures of ErpC (I18‐E76) from *B. burgdorferi* strain B31 (PDB: 4BXM), ErpP (K30‐K81) from strain B31 (PDB: 4BOB), and OspE (E40‐E89) in complex with FH20 (PDB: 4 J38). The prolonged *β*3–*β*4 loop of ErpC sterically interferes with its interaction with FH20. Residue H79 is critical for binding of complement regulators.

Solution NMR spectroscopy (PDB:2M4F) revealed that the N‐terminal segment (residues 20–41) of OspE from *Bb* s.s. N40 is unstructured and highly flexible, likely enabling the protein to move freely around its membrane anchor. The remaining residues (42–171) form a well‐defined globular domain composed of *β*‐strands and two *α*‐helices (Bhattacharjee et al., 2013). The secondary structure elements in OspE consist of four *β*‐strands followed by an *α*‐helix (*β*1–*β*2–*β*3–*β*4–*α*1–*β*5–*β*6–*β*7–*β*8–*α*2) (Figure 7a). The tetrads of *β*1–*β*4 and *β*5–*β*8 form two anti‐parallel *β*‐sheets oriented perpendicular to each other, creating a squashed asymmetric *β*‐barrel. This barrel is stabilized by hydrogen bonds between *β*1 and *β*8. The differences in lengths and shapes of the *β*‐strands contribute to the non‐classical geometry of the OspE *β*‐barrel, with strands *β*1, *β*2, and *β*6 being much longer (10, 12, and 11 residues, respectively), while the shortest strands, *β*5 and *β*8, consist of only six residues. Additionally, strands *β*2 and *β*6 are highly twisted, further contributing to the unique structural configuration of OspE (Bhattacharjee et al., 2013) (Figure 7a).

The OspE structures in solution NMR and in crystal complex with FH19‐20 (PDB: 4J38) are very similar (Bhattacharjee et al., 2013). X‐ray crystallography also demonstrates that the N‐terminus is unstructured and that OspE binds to the FH19 and FH20 domains of FH, with the complex stabilized by multiple interactions (Figure 7a) (Bhattacharjee et al., 2013). Specifically, R1182 from FH domain 20 forms an ion triplet with OspE residues E68 and D73, creating a tripartite interaction facilitated by a sulfate group, resulting in the formation of the interaction triplet R1182–SO_4_–R66 (Figure 7b). Additionally, R1182 forms an intramolecular ion pair with E1198, further enhancing the stability of the complex (Figure 7b) (Bhattacharjee et al., 2013). Nine OspE residues were identified as forming hydrogen bonds with FH modules 19 and 20 in the crystal structure (Bhattacharjee et al., 2013). Six were found to be identical across all FH‐binding proteins in the Erp family, while two can be conservatively mutated (from Asp to Glu and from Ser to Thr) (Bhattacharjee et al., 2013). Interestingly, the heparin and OspE binding sites on FH19/20 appear to overlap (Bhattacharjee et al., 2013; Meri et al., 2013).

Erp proteins (ErpA, ErpC, ErpP) are additional FH binding proteins that share over 80% sequence identity with OspE (Sung et al., 2000). ErpA and ErpP interact with both FH and FH‐related proteins (FHR‐1, FHR‐2, and FHR‐5), whereas ErpC binds only to FHR proteins and does not interact with FH (Hammerschmidt et al., 2012; Haupt et al., 2007). X‐ray resolved structures reveal a high structural similarity among the triplet OspE, ErpC, and ErpP (Figure 7c). Despite the similarities in the tertiary structure and arrangement of secondary structures, some variations have been described in the loop regions connecting strands *β*3 and *β*4 (Brangulis et al., 2015). Compared to OspE and other Erp proteins, the loop in ErpC is elongated by eight residues. Aligning the x‐ray structures of ErpC and the OspE‐FH19 + 20 complex locates the loop near the FH binding interface (Figure 7c). The extended loop of ErpC is thought to sterically hinder the ErpC‐FH interaction (Bhattacharjee et al., 2013; Brangulis et al., 2015; Caesar, Wallich, et al., 2013). A mutational non‐structural study on Erp proteins identified a single amino acid, H79 (H94 in ErpP), as essential for ligand binding (Figure 7c) (Seling et al., 2010). A histidine‐to‐arginine mutation in the recombinant protein resulted in complete loss of FH and FHR‐1 binding (Seling et al., 2010), supposedly due to steric clashes with the neighboring residues (Brangulis et al., 2015).

## 3 TREPONEMA PALLIDUM

### 3.1 Tp0751

**FIGURE 8:**
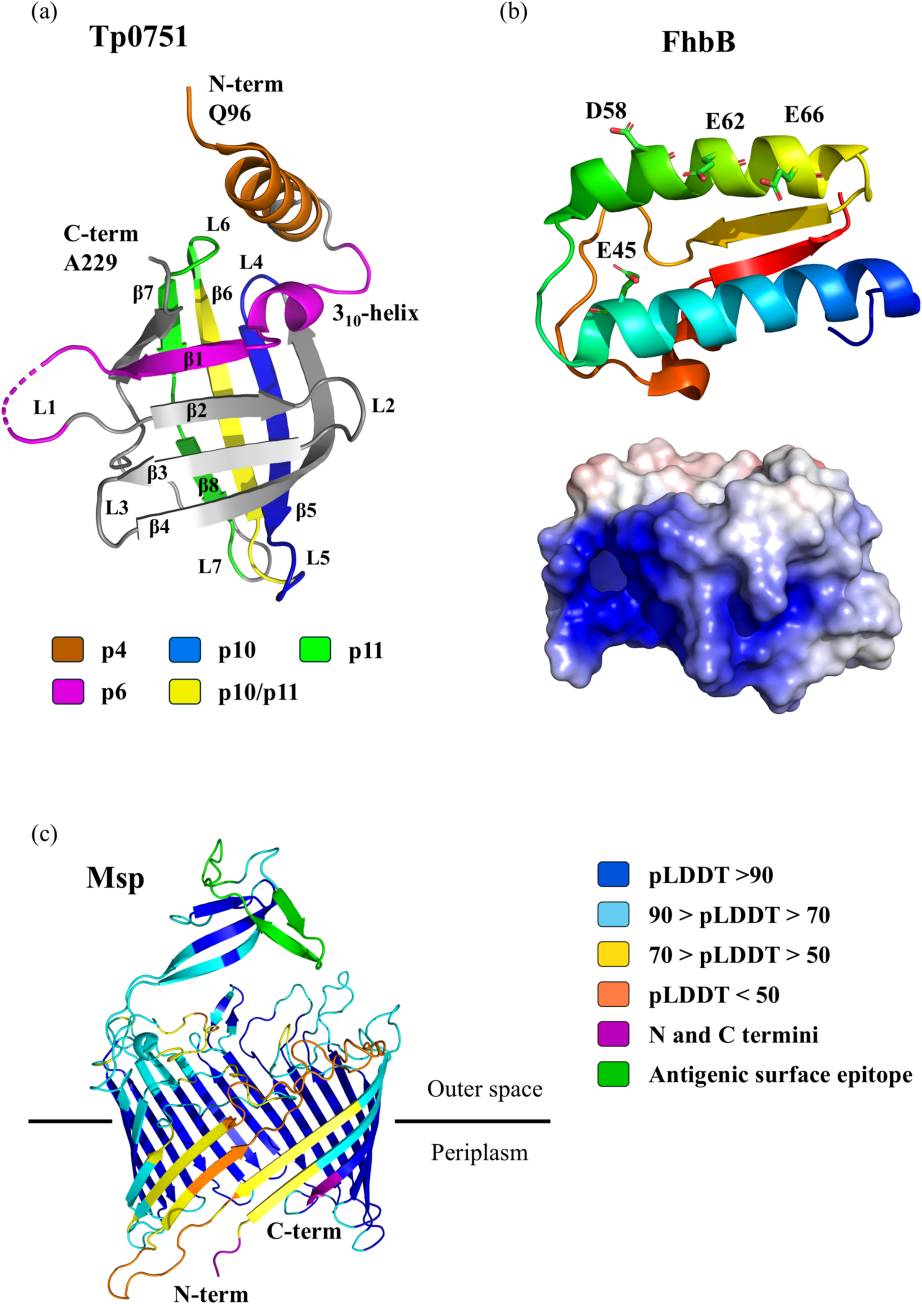
Structural models of *Treponema* proteins and their interactions. (a) X‐ray crystal structure of the ordered segment (Q96‐A229) of *T. pallidum* Tp0751 (PDB:5JK2) is shown, with laminin‐binding peptides (p4–p11) highlighted in different colors. *β*‐strands and loops (L1–L7) are numbered sequentially from the N‐terminus. The dotted line represents the partially disordered region. (b) X‐ray crystal structure of *T. denticola* FhbB (PDB: 3R15) is shown as a cartoon model, with labeled residues involved in the interaction with factor H. The electrostatic potential, calculated using the advanced Poisson–Boltzmann Solver PyMOL plug‐in, is mapped onto the surface model in a color gradient ranging from −5 to +5 kBT.e^−1^, with negative charges in red and positive charges in blue. (c) The AlphaFold‐predicted structural model of the Major Outer Sheath Protein (Msp) from *T. denticola* (UniProt accession number Q56256, without 20 AA long signal sequence). The prediction confidence is indicated by the predicted local distance difference test (pLDDT), which is represented on a scale from 0 to 100.

Tp0751 mediates adhesion of *Tp* to components of the ECM such as laminin, fibronectin, and fibrinogen (Cameron, 2003; Cameron et al., 2008). Tp0751 adopts a compact *β*‐barrel structure with attached N‐terminal helical elements (Figure 8a). In the x‐ray diffraction data (PDB: 5JK2), the absence of electron density for residues S78 to T95 indicates disorder in this region. After the first ordered residue, Q96, an *α*‐helix spans from P98 to H113, followed by a short 3_10_‐like helix formed by L119 to L123. The remainder of the protein forms an eight‐stranded anti‐parallel *β*‐barrel with a +1 topology (Parker et al., 2016).

Structure comparisons show similarity between Tp0751 and the lipocalin family of proteins (Parker et al., 2016), which are characterized by an eight‐stranded *β*‐barrel and short conserved regions (SCR1, SCR2, and SCR3) involved in fatty acid binding (Flower et al., 2000). Tp0751 shares the *β*‐barrel fold but lacks the typical N‐ and C‐terminal layouts and most conserved lipocalin features. Specifically, its SCR1 consists of G125 and W127, located within the 3_10_‐like helix and the first *β*‐strand, whereas its SCR3 includes R226 in the last *β*‐strand, forming a hydrogen bond with A126 near the 3_10_‐like helix (Parker et al., 2016). Unlike lipocalins, Tp0751 lacks a functional hydrophobic binding pocket due to spatial differences in the surrounding loops, particularly loop 7 (Figure 8a), which contains polar residues that cap the hydrophobic core (Parker et al., 2016).

A short Tp0751 peptide library, comprising overlapping sequences, was created to assess binding to laminin and other ECM components like fibrinogen, fibronectin, and collagen types I and IV (Cameron et al., 2005; Parker et al., 2016). Laminin‐binding experiments identified key residues in three peptides: p4 (P98, V99, Q100, T101), p6 (W127, I128), and p10 (T182, A183, I184, S185) (Figure 8a). However, mutation analysis showed that the residues in p6 are not essential for laminin binding. Inhibition assays further demonstrated that peptides p4, p6, and p10 effectively reduced *Tp* attachment to laminin‐coated surfaces (Parker et al., 2016). Beyond laminin, peptides p4, p6, and p11 exhibited significant binding to fibrinogen, fibronectin, and collagen types I and IV. Peptide p10 bound specifically to fibronectin, fibrinogen, and collagen type IV, but not to collagen type I. Additionally, p10 reduced the binding of Tp0751‐expressing *Bb* to human endothelial cells (Parker et al., 2016).

## 4 TREPONEMA DENTICOLA

### 4.1 FhbB

Factor H‐binding protein B (FhbB) is a small, yet critical virulence factor produced by *Td*, a spirochete associated with periodontal disease. This surface‐exposed lipoprotein is instrumental in immune evasion by interacting with the complement regulator FH (McDowell et al., 2007; Simonson et al., 1988).

The crystal structure of FhbB at a resolution of 1.7 Å (PDB:3R15) revealed a unique fold comprising *α*1–*α*2–*β*1–*α*3–*β*2 secondary structures with a well‐defined hydrophobic core (Figure 8b). The *β*1–*α*3–*β*2 bundle forms the positively charged side of the protein, while the opposing negatively charged side is formed by the *α*1 and *α*2 helices. Helices *α*1 and *α*2 are connected by a loop spanning residues K47 to P52, while a second loop, linking *β*1 and *α*3, is located between residues K77 and K88. These loops are interconnected by a network of polar interactions, contributing to structural integrity. Structural alignment using DALI (Holm & Rosenström, 2010) classifies the FhbB fold as unique. Its helical architecture, stabilized by a hydrophobic core and a single hydrogen bond between H34 and Y72, provides exceptional thermostability up to 90°C. Under laboratory conditions, FhbB forms a weak dimer at high concentrations. The dimeric interaction occurs along the *α*2 helices in an anti‐parallel orientation within the asymmetric unit. However, the small interface area, presence of interfacial water molecules, lack of hydrogen bonds, and minimal van der Waals interactions indicate that the dimer is not relevant for FhbB in its native state, and FhbB likely functions as a stable monomer in vivo (Miller et al., 2012).

The interaction between FhbB and FH was investigated by site‐directed mutagenesis. Four residues—E45, D58, E62, and E66—located on the negatively charged *α*1–*α*2 side of the protein are critical for FH binding (Figure 8b) (Miller et al., 2012). Mutating these residues reduces FH binding significantly, while mutations on the opposite side of the protein have no effect. Intramolecular hydrogen bonds within FhbB also play a crucial role in FH binding by stabilizing the negatively charged electrostatic environment required for the interaction (Miller et al., 2012). Two hydrogen bonds are formed within *α*1 and turn1, while another H‐bond links turn1 and turn2. Residues such as I63, L68, F96, and several non‐polar, non‐surface‐exposed residues at the C‐terminus indirectly influence FH binding (McDowell et al., 2009). Although these residues do not directly participate in the interaction, they contribute to the stability of the protein core, which indirectly supports ligand binding (McDowell et al., 2005; Miller et al., 2012).

### 4.2 Major outer sheath protein

Msp is a pore‐forming outer membrane protein that binds fibronectin and laminin, playing a key role as a virulence factor in the development of periodontal disease (Egli et al., 1993; Goetting‐Minesky et al., 2022; Haapasalo et al., 1992). While no complete molecular structure of Msp has yet been determined, a combination of experimental immunotopological data and modeling approaches such as the AlphaFold2‐multimer pipeline has provided predictions of its structural properties. These analyses indicate that Msp adopts a trimeric form, with N‐ and C‐termini positioned to promote trimerization via inter‐domain interactions (Goetting‐Minesky et al., 2022). This modeled structure supports immunodetection experiments, where antibodies against native Msp show higher affinity for oligomeric forms (Goetting‐Minesky et al., 2022).

Initial analysis of the Msp protein sequence suggested that it forms a *β*‐barrel structure spanning the outer membrane (Figure 8c) (Fenno et al., 1996). Later studies revealed that Msp consists of two conserved domains, MOSP‐N and MOSP‐C (Anand et al., 2013). Orthologous domains are also found in TrpC protein from *Tp* (Anand et al., 2015). The MOSP‐C domain forms an amphiphilic *β*‐barrel structure embedded in the membrane and contains surface‐exposed antigenic epitopes (Godovikova et al., 2018). In contrast, the MOSP‐N domain is a hydrophilic *α*‐helical structure that extends into the periplasmic space (Anand et al., 2013). Mutation studies in both the N‐ and C‐terminal *β*‐strands of Msp have demonstrated their essential role in outer membrane oligomerization and protein stability (Goetting‐Minesky et al., 2022). Partial cleavage of Msp has been described in some studies, which identified the release of a proteinase K‐resistant 25 kDa peptide containing an antigenic surface epitope (Figure 8c) (Anand et al., 2013; Godovikova et al., 2018; Puthenveetil et al., 2017).

## 5 LEPTOSPIRA

### 5.1 Leptospiral immunoglobulin‐like proteins

**FIGURE 9:**
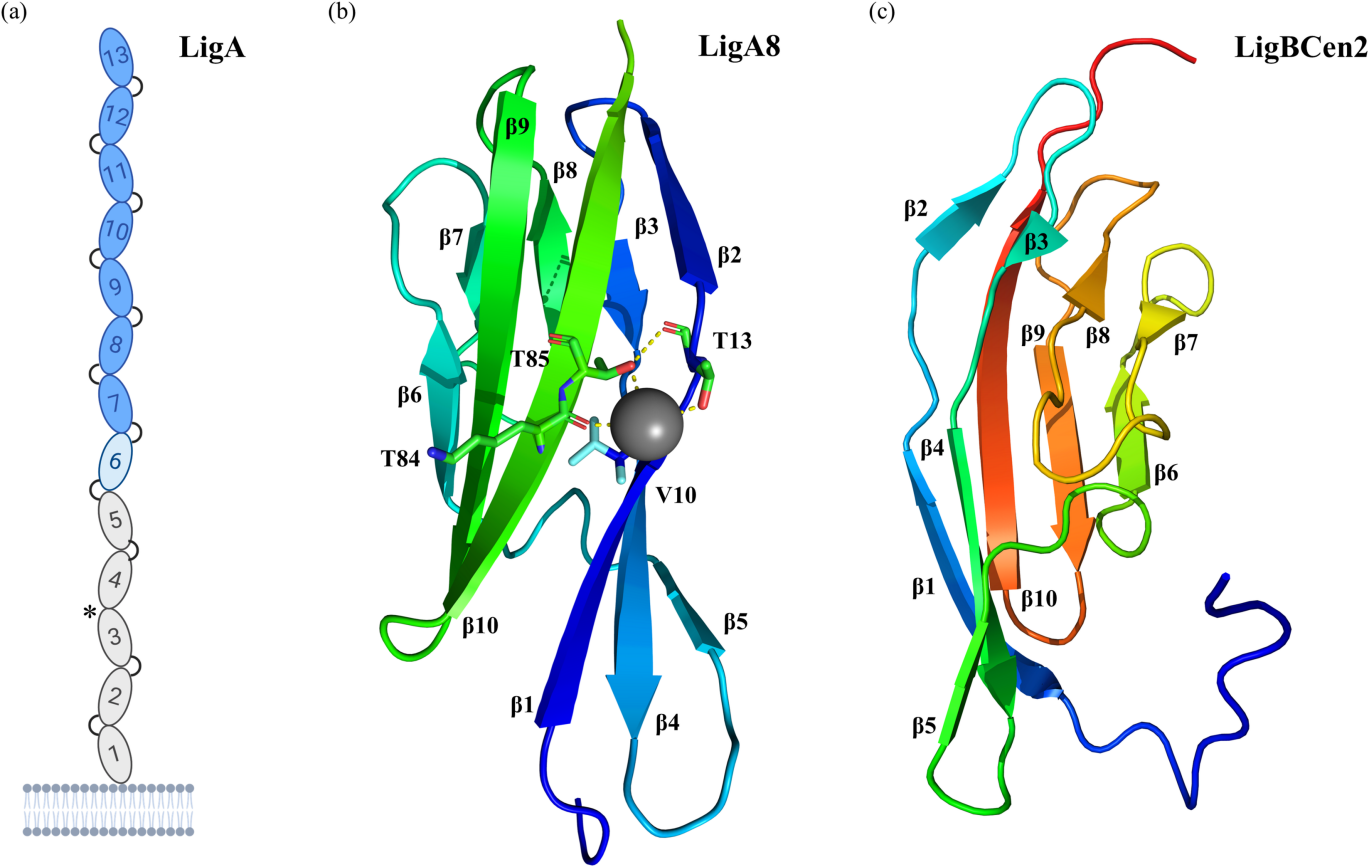
(a) Schematic representation of the LigA protein anchored to the outer membrane of *Leptospira*. LigA domains are numbered, with constant regions (domains 1–5) shown in gray and variable regions (domains 7–13) in blue. The boundary within domain 6 is marked in light blue. Connecting arcs indicate salt bridges between domains, while the absence of a salt bridge between LigA3 and LigA4 is marked with an asterisk. (b) X‐ray crystal structure of LigA domain 8 (PDB: 8GYR; domain 9 was removed from the structure), showing the main residues involved in interactions with the Ca^2+^ ion. (c) Solution NMR structure of LigBCen2 (PDB: 2MQG), which contains part of the 11th and the entire 12th domain. *β*‐strands are numbered sequentially from the N‐terminus.

Leptospiral immunoglobulin‐like (Ig‐like) domain proteins such as LigA and LigB are members of the *Leptospira* family of multidomain surface‐exposed proteins, which are among the most conserved and extensively studied proteins in pathogenic *Leptospira* (Haake & Matsunaga, 2021). *Leptospira* utilizes Lig proteins to facilitate interactions with a variety of specific host proteins, including those involved in circumventing innate immune defenses (Haake & Matsunaga, 2021). Strong binding to fibronectin has been demonstrated using a fragment containing the C‐terminal Ig‐like domains (domains 7–12) of both LigA (Figure 9b) and LigB (Choy et al., 2007; Lin et al., 2010). In contrast, the N‐terminal Ig‐like domains shared by LigA and LigB did not interact with fibronectin (Choy et al., 2007; Lin & Chang, 2008). The C‐terminal Ig‐like domains of LigB also showed moderate affinity for collagen I, collagen IV, and laminin, while the C‐terminal Ig‐like domains of LigA displayed weak binding to collagen and no detectable interaction with laminin (Choy et al., 2007). *Leptospira* also employs LigA and LigB to recruit host complement regulators, such as factor H and C4b‐binding protein, thereby enhancing resistance to complement‐mediated attack (Castiblanco‐Valencia et al., 2012).

LigA consists of 13 homologous tandem repeats of Ig‐like domains (Figure 9b), each approximately 90 AAs in length. These domains are connected by short 4–5 AA linkers. The AA sequence in the first six domains, along with half of the seventh domain, is highly conserved, while the remaining domains show greater variability. LigB is a related protein containing 12 Ig‐like domains, with the first six domains and half of the seventh one conserved like in LigA. However, LigB features a 772 AAs C‐terminally largely disordered extension (Matsunaga et al., 2003).

The crystal structure of LigA domains 8 (Figure 9a) and 9 (PDB: 8GYR, resolution 1.87 Å) contains two LigA8‐9 molecules in the asymmetric unit. This structure consists of 21 *β*‐strands organized into six *β*‐sheets. The LigA8 domain comprises 10 *β*‐strands arranged in an anti‐parallel pattern (Kumar et al., 2023). This domain follows the classical Ig‐like architecture, with anti‐parallel *β*‐strands folded into two *β*‐sheets that form a hydrophobic core (Raman et al., 2013; Wang, 2013). In LigA8, three sheets are formed: Sheet A consists of strands 1, 4, and 5; Sheet B includes strands 3, 7, and 8; and Sheet C is composed of strands 2, 6, 9, and 10. The LigA9 domain adopts a similar fold, with strand 19 (equivalent to strand 9 in LigA8) followed by two shorter strands, resembling strand 10 in LigA8. In LigA9, Sheet A is formed by strands 11, 14, and 15; Sheet B by strands 13, 17, and 18; and Sheet C by strands 12, 16, 19, 20, and 21 (Kumar et al., 2023). Notably, a proline peptide bond in the turn connecting the first and second *β*‐strands in both domains adopts a cis conformation (Kumar et al., 2023) introducing a kink, which is a conserved feature in many Ig‐like proteins (Wang, 2013).

The inter‐domain arrangement of the LigA protein units is crucial for interactions with the ECM. The linker between the domains is flexible, with the angle between the LigA8 and LigA9 domains varying around 124°. The orientation and distance between these domains are influenced by a salt bridge formed between a lysine residue in the linker between strands 2 and 3 of LigA8 and an aspartate in the linker between strands 4 and 5 of LigA9. This salt bridge is conserved in all LigA domains, except for LigA3 and LigA4. No salt bridge has been proposed between the LigA1 and LigA2 domains. Structure studies suggest LigA architecture is organized as a long chain of domains connected in a zigzag pattern (Figure 9b) (Kumar et al., 2023).

Studies have observed Ca^2+^ binding in LigA domains and its role in enhancing interactions with the ECM (Raman et al., 2010). In LigA8 (Figure 9a), a high electron density suggests an interaction with Ca^2+^ ions. The Ca^2+^ ion coordinates with both backbone atoms and side chains of AAs, interacting with Val10 and Thr13 from strand 1 and the strand 1–2 linker, respectively. Lys84 and Thr85 of strand 10 are also involved in Ca^2+^ binding (Kumar et al., 2023). Comparing the NMR solution structure of the LigA4 domain (PDB 2N7S) to the crystal structures of the LigA8 and LigA9 domains revealed RMSD values of 2.64 Å for LigA8 and 2.42 Å for LigA9. Major structural differences between LigA4 and LigA8 were observed in the regions connecting strands 6 and 7, with similar variations noted in LigA9. Another notable variation in LigA8 occurred in the region connecting strands 5 and 6 (Kumar et al., 2023; Mei et al., 2015b). NMR CSP experiments of LigA4 in the presence of Ca^2+^ demonstrated resonance shifts at many residues. Among these, V10, S11, T13, T16, V17, A18, K19, E23, N24, and F25 were identified as forming a negatively charged region that facilitates Ca^2+^ binding (Kumar et al., 2023; Mei et al., 2015b).

The crystal structure of LigA domains (PDB:8GYR) was compared to the NMR solution structure of the LigB12 domain (PDB:2MQG), revealing RMSD differences of 1.8 and 1.5 Å, respectively. In both structures, a conserved lysine residue in the loop interacts with asparagine from the preceding domain in a manner similar to the interaction observed between LigA8 and LigA9. Additionally, LigB12 contains a cis‐proline bond at the same position as in LigA (Kumar et al., 2023; Ptak et al., 2014).

The central variable region of LigB, known as LigBCen2, which includes the LigB12 domain with additional AA residues at both its N‐ and C‐termini, has also been shown to bind Ca^2+^ and modulate fibronectin binding (Lin et al., 2008). The solution NMR structure of LigBCen2 (PDB: 2MQG) revealed that the protein adopts a canonical Ig‐like structural pattern, forming a *β*‐sandwich composed of three sheets and 10 strands, similar to other Lig proteins (Mei et al., 2015a). Isothermal calorimetry titrations determined a Ca^2+^ binding affinity with a *K*_D_ of 0.3 μM. NMR CSPs were observed in residues at both the N‐ and C‐termini, as well as in the loop region between strands 7 and 8. The binding site for Ca^2+^ in the 7–8 loop of LigBCen2 was identified as residues S66, E67, T68, and K69 (Mei et al., 2015a).

## 6 CONCLUSION

Although the functional role of many spirochetal proteins in pathogenesis has been identified (Coburn et al., 2021; Strnad et al., 2023), understanding of the structural features responsible for their biological activity remains very limited. With the continued progress of high‐resolution structure determination techniques such as x‐ray crystallography, NMR, and cryo‐electron microscopy, researchers can envisage the 3D structure of larger and larger molecules as well as supramolecular entities and thus identify key binding sites or active regions crucial for pathogen survival and host interactions. Recent methodological advances have also improved our ability to characterize disordered regions and their dynamics, which determine their function in pathogenic processes.

**FIGURE 10:**
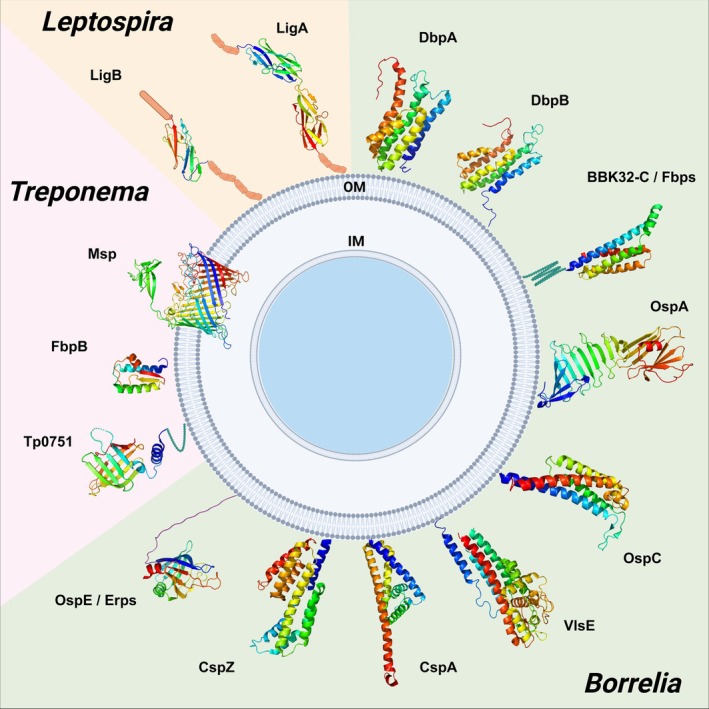
Structural overview of adhesive and immunomodulatory surface‐exposed proteins in spirochetes.

Remarkable mechanistic variability has been observed in the binding of spirochetal surface‐exposed proteins to host ligands (Figure 10). For example, Dbps interact with highly negatively charged GAG ligands primarily through electrostatic interactions. The structural variability and dynamic properties of the Dbp linker section, including differences in its length and the presence of a helical structure, along with the flexible C‐terminus, modulate binding affinity across different *Bb* genospecies (Benoit et al., 2011; Morgan et al., 2015; Morgan & Wang, 2015). Notably, the apparent flexibility observed in unstructured or poorly conserved regions may not reflect inherent disorder, but rather a lack of structural information under the current experimental conditions. It is plausible that these regions adopt defined conformations in the context of the lipidated protein or when embedded within a membrane or membrane‐mimetic system. As such, future structural studies in more native‐like environments may reveal previously unrecognized order in these segments (Davey, 2019).

Structural polymorphism has been found in the binding sites of many proteins discussed in this review. For instance, the complement regulatory fragments of LD *Borrelia* BBK32‐C and RF *Borrelia* FBPs contain two major polymorphic domains (Booth et al., 2022; Roy et al., 2023; Xie et al., 2019). Similarly, factor H‐binding proteins such as CspZ and Erps show variability in the loops involved in interactions (Bhattacharjee et al., 2013; Brangulis et al., 2015; Marcinkiewicz et al., 2023). In contrast to Dbps, the fibronectin‐binding activity of BBK32 relies on inter‐residual hydrophilic interactions, salt bridges, and water‐mediated interactions (Harris et al., 2014). OspA adopts a unique secondary structure with globular domains at both ends, resembling a dumbbell. However, the central *β*‐sheet lacks the typical hydrophobic core structure found in similar proteins (Koide et al., 2000; Pham et al., 1998). The treponemal Tp0751 protein appears to share a structural pattern similar to that of OspE and Erps proteins due to its perpendicular *β*‐sheet fold with attached short helices. However, the *β*‐barrel fold of Tp0751 is similar to the structure found in the fatty acid‐binding lipocalin protein family. Despite this similarity, small structural differences in Tp0751 result in a hydrophobic core that does not function like the lipocalin proteins (Flower et al., 2000; Parker et al., 2016).

Blocking adhesive bacterial molecules represents an innovative and promising antibacterial strategy (Asadi et al., 2019; Lin et al., 2017). In spirochetes, adhesins are distributed across the entire cell outer surface and function without the requirement to be presented on pili or fimbriae. The accessibility of outer surface molecules makes them prime targets for highly specific therapeutic interventions since they are directly exposed to the external environment. This exposure allows drugs to interact with these molecules without crossing complex biological barriers. By targeting adhesive molecules, it is possible to disrupt bacterial attachment, making it difficult for bacteria to colonize host tissues (Firon et al., 1987; Ofek et al., 2003; Simon et al., 1997). This approach is very effective in preventing biofilms that are notoriously resistant to antibiotics and immune response (Bernal‐Mercado et al., 2020; Grooters et al., 2024). Additionally, blocking the surface proteins of pathogens that interact with immunomodulatory host proteins, such as FH, may be crucial for enhancing immune responses. Many pathogens often use such interactions to evade the immune system by inhibiting the complement system (Agarwal et al., 2010; Castiblanco‐Valencia et al., 2012; Haupt et al., 2008; Kraiczy et al., 2006; Kunert et al., 2007). By preventing these bindings, the host can activate complement responses more effectively, improve opsonization, and eliminate pathogens.

Structure‐based drug design is a powerful approach that utilizes detailed knowledge of molecular structures to develop targeted therapeutic agents (Batool et al., 2019). Compared with traditional drug discovery methods (classical or forward pharmacology), rational drug design is more efficient and cost‐effective. By leveraging structural insights, drugs can be precisely designed to bind specific molecular sites, thereby inhibiting critical functions such as host cell attachment. Several drugs that are effective against pathogens have been discovered using structure‐based drug design (Anderson, 2003; Batool et al., 2019; Rutenber & Stroud, 1996; Wlodawer & Vondrasek, 1998). The most notable success has been the development of FDA‐approved drugs that inhibit HIV‐1 (Wlodawer & Vondrasek, 1998). Structure‐based vaccine design using borrelial CspZ has demonstrated significant improvements in immunization studies by unmasking protective epitopes, thereby enhancing vaccine efficacy (Brangulis et al., 2025).

Currently, the therapeutic treatment of LD and other spirochetal diseases is limited to broad‐spectrum systemic antibiotics (Strnad et al., 2020). These antibiotics also affect the natural bacterial colonization of the skin, mucous membranes, and the gastrointestinal tract. They can lead to a disturbance in the bacterial balance, resulting in the selection of resistant pathogenic species, even those unrelated to the target. Blocking surface molecules associated with virulence would reduce the selective pressure for developing antibiotic resistance, which is a major advantage of adhesion blockers. Because such therapeutic agents do not kill bacteria outright, but rather prevent them from establishing infection, the target bacteria may be less likely to develop resistance (Levy, 2002). Additionally, this strategy could be used in combination with traditional antibiotics, making bacteria more vulnerable to treatment by disrupting adhesion and biofilm formation. Challenges for successful drug design remain, in particular, ensuring specificity to avoid the impact on beneficial bacteria and achieving effective delivery of surface protein blockers to infection sites. Despite these hurdles, adhesion inhibitors could have a wide range of applications, from coating medical devices to prevent infections, to topical treatments for parasite‐inflicted lesions, as well as respiratory and gastrointestinal infections.

## AUTHOR CONTRIBUTIONS

**Libor Hejduk:** Writing – original draft; writing – review and editing; visualization. **Norbert Müller:** Writing – review and editing; funding acquisition. **Adriana Rathner:** Supervision; writing – review and editing. **Ján Štěrba:** Project administration. **Shang‐Cheng Hung:** Supervision; funding acquisition. **Chia‐Lin Chyan:** Supervision. **Ryan O. M. Rego:** Supervision. **Martin Strnad:** Conceptualization; supervision; writing – original draft; writing – review and editing; funding acquisition.

## Data Availability

Data sharing is not applicable to this article as no new data were created or analyzed in this study.
